# Glycolipid dynamics in generation and differentiation of induced pluripotent stem cells

**DOI:** 10.1038/srep14988

**Published:** 2015-10-19

**Authors:** Takuma Ojima, Eri Shibata, Shiho Saito, Masashi Toyoda, Hideki Nakajima, Mayu Yamazaki-Inoue, Yoshitaka Miyagawa, Nobutaka Kiyokawa, Jun-ichiro Fujimoto, Toshinori Sato, Akihiro Umezawa

**Affiliations:** 1 National Research Institute for Child Health and Development, Tokyo, 157-8535, Japan; 2 Department of Biosciences and Informatics, Keio University, Kanagawa, 223-8522, Japan; 3 Department of Research Team for Geriatric Medicine, Tokyo Metropolitan Institute of Gerontology, Tokyo, 173-0015, Japan

## Abstract

Glycosphingolipids (GSLs) are glycoconjugates that function as mediators of cell adhesion and modulators of signal transduction. Some well-defined markers of undifferentiated human embryonic stem cells (hESCs) and human induced pluripotent stem cells (hiPSCs) are glycoconjugates, such as SSEA-3, SSEA-4, TRA-1-60 and TRA-1-81. However, Comprehensive GSL profiles of hiPSCs have not yet been elucidated. The global images of GSLs from the parental cells, hiPSCs, and differentiated cells revealed that there are parental cell-independent specific glycolipids, including Globo H (fucosyl-Gb5Cer) and H type1 antigen (fucosyl-Lc4Cer) that are novel markers for undifferentiated hiPSCs. Interestingly, undifferentiated hiPSCs expressed H type 1 antigen, specific for blood type O, regardless of the cells’ genotypes. Thus, in this study, we defined the dynamics of GSL remodeling during reprogramming from parental cell sets to iPSC sets and thence to iPSC-neural cells.

When the technology to generate human iPS cells (hiPSCs) first became available^1^,^2^, immediate attention was placed on their potential for use in cell-based transplantation. Both iPSCs (differentiated *in vitro*) and embryonic stem cells (ESCs) can provide an unlimited source of useful cell types for transplantation. The use of hiPSCs is desirable because they lack the substantial ethical concern of cellular origin that plagues ESCs. The fact that hiPSCs are autologous for patients is another advantage in transplantation. However, one of the major drawbacks for transplantation of iPSCs is their labile/variable state due to epigenetic dynamics during cultivation and their carcinogenic potential due to oncogene infection^3^. Soon after hiPSC technology was developed, researchers began to realize an additional and possibly greater value for the cells to help elucidate the causes of disease. hiPSCs can be generated from skin biopsies or blood samples of patients, and can differentiate *in vitro* into cell types that are not easily accessible from patients.

The glycans expressed on the cell membrane differ among cell lines, and participate in development, differentiation, activation, inflammation, and malignant transformation^4^,^5^,^6^. Therefore, glycan profiling, in addition to genomic and epigenetic profiling, may provide valuable information about the signal transduction pathways during these events, and in fact has already shown promise in the fields of reproductive medicine and oncology^7^,^8^. Antibodies are commonly employed to recognize glycans in cells, and lectins, which recognize specific glycan epitopes, have been used for blood-group typing, tissue staining, lectin-probed blotting and flow cytometry. Recently, a lectin microarray enabled discriminate glycan profiling between different cell lines by ultrasensitive detection of multiplex lectin-glycan interactions^9^. In addition to the use of antibodies and lectins, the saccharide primer method has been used to screen essential cell-surface carbohydrates^10^,^11^. Biochemical approaches such as liquid chromatography-tandem mass spectrometry (LC-MS) have also been used to analyze carbohydrate structures for identification of cell types and for evidence of transformation^12^,^13^,^14^. The comprehensive analysis with LC-MS also revealed specific glycan structures in pluripotent stem cells and somatic cells^15^.

Stem cells have the ability to divide, self-renew and to differentiate into various cell types. Stem cells have varying degrees of differentiation potential: (a) totipotency (the ability to form the embryo and placental cells), as seen in fertilized eggs (zygotes); (b) pluripotency (the ability to differentiate into almost all cells that arise from the three germ layers), as found in hESCs and hiPSCs; (c) multipotentiality (the capability of producing a limited range of differentiated cell lineages upon their location), as demonstrated by most tissue-based stem cells; and (d) unipotentiality (the ability to generate one cell type), as exhibited by epidermal stem cells and the spermatogonial cells of the testis. That is, a hierarchy of stem cells exists. In addition, ESCs show variation in differentiation propensity. iPSCs, another type of pluripotent stem cell, have been generated from somatic cells of different origins by retroviral transduction of four transcription factors^1^,^2^. The established iPSCs have a wider variety of differentiation ability and gene expression than ESCs. However, a small proportion of these stem cells sometimes show spontaneous differentiation during serial passage. Therefore, in order to realize the full potential for iPSCs in cell therapy and drug discovery, it is necessary to monitor the status of these stem cells and to define their exact stage during processes of growth and/or differentiation.

Carbohydrate epitopes are often used as markers for definition and characterization of stem cells. Stage-specific embryonic antigens such as SSEA-3, SSEA-4, TRA-1-60 and TRA-1-81 are also used as markers for the undifferentiated state of human ESCs (hESCs) and hiPSCs^1^. Glycosphingolipids (GSLs) expressed in hESCs have been examined by immunofluorescence, flow cytometry and mass spectrometry^16^,^17^. GSLs such as Gb5Cer (SSEA-3), sialyl-Gb5Cer (SSEA-4), fucosyl-Gb5Cer (Globo H), and IV fucosyl-Lc4Cer (H type 1 antigen), have been identified in hESCs. In this study, we investigated the hiPSC-specific GSLs that were induced and highly expressed at the earliest stages of iPSCs generation and then down-regulated upon differentiation. We propose that the glycolipid dynamics during generation and differentiation of iPSCs will lead to a better understanding of cellular reprogramming.

## Results

### Analysis of GSLs in MRC-5 cells and UtE cells

**Figure 1:**
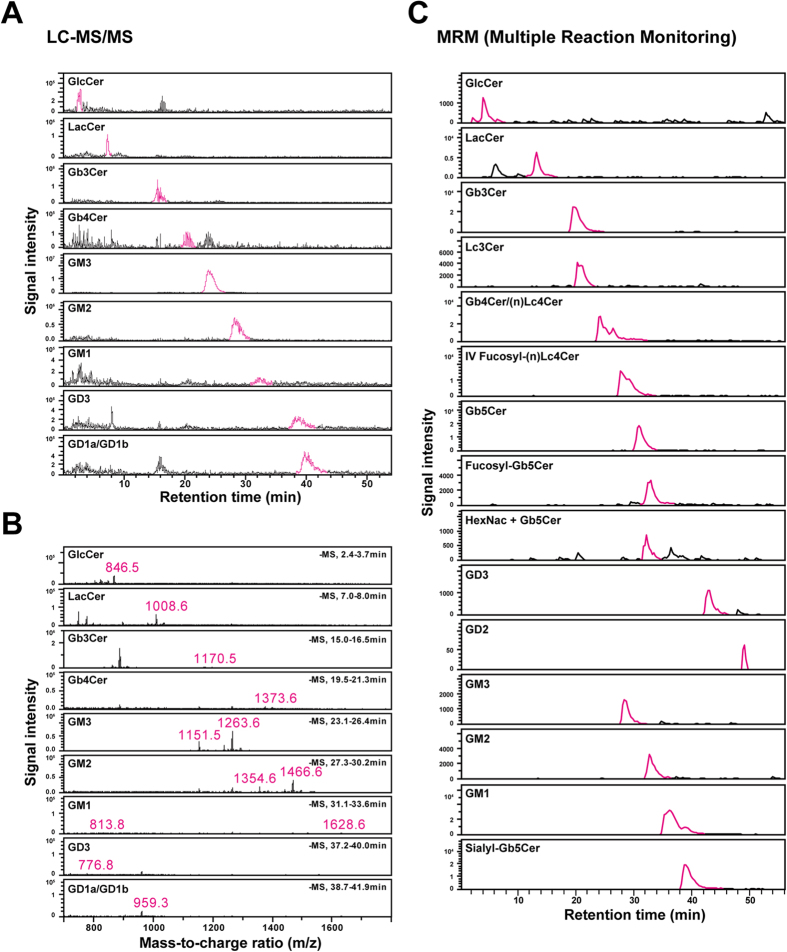
LC-MS/MS analysis on GSLs of iPS cells and their parental cells. (**A**–**C**) LC chromatogram and mass spectrum on iPS cells and parental MRC-5 cells. (**A**) LC chromatogram of MRC-5 cells. (**B**) Mass spectrum of MRC-5 cells. (**C**) LC chromatogram of MRCiPS#25 cells.

GSLs in MRC-5 and UtE cells were analyzed using LC-MS (Fig. 1A,C); the results are shown in Table 1. Though the fatty acid length of ceramide varied from C14:0 through C26:0, only the results for GSLs with C16:0 and C24:0 are indicated in Table 1. Analyses of MS/MS spectra revealed that the four neutral GSLs were deduced to be GlcCer, LacCer, Gb3Cer, Gb4Cer, and the five acidic GSLs were deduced to be GM3, GM2, GM1, GD3, and GD1a/GD1b for both MRC-5 cells and UtE cells (Supplemental Table S1A, S1B). The neutral GSLs were detected as Hex-Cer, Hex-Hex-Cer, and Hex-Hex-Hex-Cer, HexNAc-Hex-Hex-Hex-Cer by MS/MS analysis. Though the neutral GSLs have isomers depending on the variety of monosaccharide, their structures were deduced to be GlcCer, LacCer, Gb3Cer, Gb4Cer because they are major GSLs and detected even in hESCs^18^. However, since GalCer and galabiose as isomers of GlcCer and LacCer have been detected in hESCs^18^, existence of other isomers in hiPSCs could not be excluded.

### Analysis of GSLs in hiPSCs

**Figure 2:**
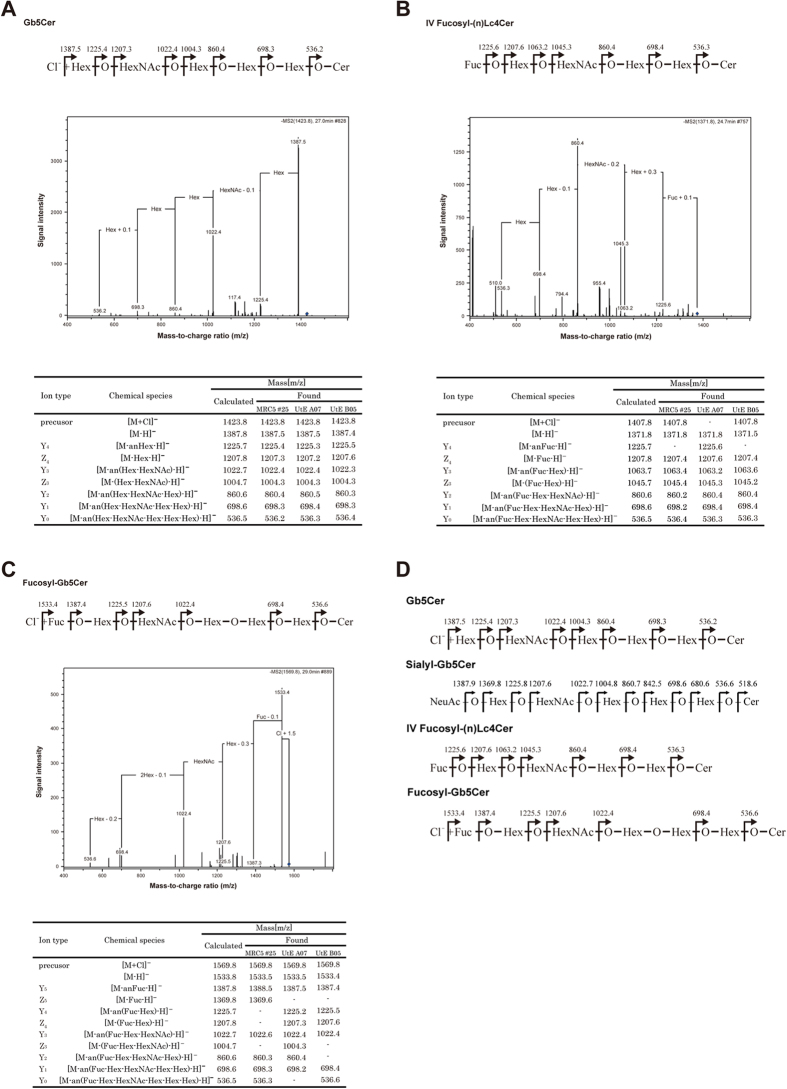
GSLs of iPS cells and their parental cells. (**A**–**C**) MS/MS spectrum of iPS cells. (**A**) Gb5Cer. (**B**) IV Fucosyl-(n)Lc4Cer. (**C**) Fucosyl-Gb5Cer. (**D**) Sequences of Gb5Cer, sialyl-Gb5Cer, V fucosyl-(n)Lc4Cer and fucosyl-Gb5Cer deduced by MS/MS analysis.

Undifferentiated hiPSCs induced from MRC-5 and UtE cells were identified by immunostaining using antibodies against SSEA-4, TRA-1-60, NANOG, OCT3/4, and SOX2 (Supplemental Figure S1). GSLs of the hiPSCs were analyzed using LC–MS. Though the fatty acid length of ceramide ranged from C16:0 through C26:0, the major fatty acids were C16:0 and C18:0. When the ceramide was d18:1/C16:0, extracted-ion chromatogram (EIC) of m/z at 734.5, 896.6, 1058.6, 1261.7, 1407.8, 1423.8, 1569.8 as [M + Cl]^−^, 1151.7, 1354.8, 1516.8, 1678.8 as [M-H]^−^ and 720.9 as [M-2H]^2−^ were detected for both MRC-iPSCs and UtE-iPSCs. Thus, two kinds of hiPSCs induced from the different parental cells showed identical GSL composition; there was no significant difference between MRC-iPSCs and UtE-iPSCs. Eight neutral GSLs and five acidic GSL were detected. By MS/MS analysis (Supplemental Table S1C and S1D), the eight neutral GSLs were deduced to be GlcCer (m/z 734.5), LacCer (m/z 896.6), Gb3Cer (m/z 1058.6), Gb4Cer/(n)Lc4Cer (m/z 1261.7), IV fucosyl-(n)Lc4Cer (m/z 1407.8), Gb5Cer (m/z 1423.8), fucosyl-Gb5Cer (m/z 1569.8). The five acidic GSLs were deduced to be GM3(m/z 1151.7), GM2 (m/z 1354.8), GM1 (m/z 1516.8), GD3 (m/z 720.9) and sialyl-Gb5Cer (m/z 1678.8). Of note, Gb5Cer, sialyl-Gb5Cer, fucosyl-Gb5Cer and IV fucosyl-(n)Lc4Cer were detected in iPSCs, but not in parental cells. The sequence of Gb5Cer deduced by MS/MS analysis was Hex-HexNAc-Hex-Hex-Hex-Cer (Fig. 2A,D). Using the same methodology, the sequence of sialyl-Gb5Cer was NeuAc-Hex-HexNAc-Hex-Hex-Hex-Cer (Fig. 2D); IV fucosyl-(n)Lc4Cer was Fuc-Hex-HexNAc-Hex-Hex-Cer (Fig. 2B,D) and the sequence of fucosyl-Gb5Cer was Fuc-Hex-HexNAc-Hex-Hex-Hex-Cer (Fig. 2C,D). Though those sequences have isomers, the structures were deduced from the MS/MS analysis and immunocytochemical analysis described later. They were major GSLs synthesized by biosynthetic pathways expressed in hESCs^16^,^17^,^18^,^19^. This is the first time that the structures for Gb5Cer, sialyl-Gb5Cer, fucosyl-Gb5Cer, and IV fucosyl-(n)Lc4Cer were identified in undifferentiated hiPSCs.

### Analysis of GSLs in NSC differentiated from hiPSCs

To investigate the alteration of GSLs during differentiation of hiPSCs, hiPSCs were differentiated to neural stem cells (NSCs) according to the standard protocol. The differentiation of MRC- and UtE-iPSCs to NSCs was confirmed by immunostaining using antibodies against SOX1, SOX2, PAX6, NESTIN, and TUJ-1 as NSC markers (Supplemental Figure S2), and OCT3/4, NANOG, SOX2, SSEA-3, SSEA-4, TRA-1-60 as iPS markers. Moreover, the expression of NSC markers such as SOX1, PAX6, HES5, NOTCH, and GPM6A were confirmed by RT-PCR. These results confirmed the successful differentiation from iPSCs to NSCs. GSLs of NSCs was analyzed by LC-MS. Though the length of the fatty acid in ceramide ranged from C16:0 through C26:0, the major fatty acids were C16:0 and C24:0. When ceramide was d18:1/C16:0, EIC of m/z at 734.5, 896.6, 1058.6, 1099.7, 1261.7, 1407.8 as [M + Cl]^−^, 1151.7, 1354.8, 1516.8 as [M-H]^−^, and 720.9, 822.4, 903.5 as [M-2H]^2−^ were detected. By MS/MS analysis (Supplemental Table S2A, S2B), the six neutral GSLs were deduced to be GlcCer (m/z 734.5), LacCer (m/z 896.6), Gb3Cer (m/z 1058.6), Lc3Cer (m/z 1099.7), (n)Lc4Cer (m/z 1261.7), III fucosyl-(n)Lc4Cer (m/z 1407.8); and the six acidic GSLs were deduced to be GM3 (m/z 1151.7), GM2 (m/z 1354.8), GM1 (m/z 1516.8), GD3 (m/z 720.9), GD2 (m/z 822.4), GD1a /GD1b (m/z 903.5). The sequence of III fucosyl-(n)Lc4Cer deduced by MS/MS analysis is Hex-(Fuc-)HexNAc-Hex-Hex-Cer. The Gb5Cer, sialyl-Gb5Cer, fucosyl-Gb5Cer, and IV fucosyl-(n)Lc4Cer expressed in hiPSCs were not detected in NSCs, while III fucosyl-(n)Lc4Cer was detected. There was no significant difference between the NSCs differentiated from MRC-iPSCs and UtE-iPSCs.

### Analysis of GSL in embryoid body (EB) outgrowth differentiated from iPSCs

The alteration of GSLs upon differentiation from hiPSCs to EB outgrowth was also investigated. Differentiation of MRC-iPSCs and UtE-iPSCs to tridermic EB cells was confirmed by immunostaining with antibodies against Tuj-1, αSMA and AFP (Supplemental Figure S3A). GSLs of EB outgrowth were analyzed by LC-MS. Though the length of fatty acid in ceramide varied from C16:0 through C26:0, major fatty acids were C16:0 and C24:0, and were similar to the fatty acids found in NSCs. When ceramide was d18:1/C16:0, EIC of m/z at 734.5, 896.6, 1058.6, 1099.7, 1261.7, 1407.8 as [M + Cl]^−^, and 1151.7, 1354.8, 1516.8 as [M-H]^−^, and 720.9, 822.4, 903.5 as [M-2H]^2−^, were detected. By MS/MS analysis (Supplemental Table S2C, S2D), the six neutral GSLs were deduced to be GlcCer (m/z 734.5), LacCer (m/z 896.6), Gb3Cer (m/z 1058.6), Lc3Cer (m/z 1099.7), (n)Lc4Cer (m/z 1261.7), III fucosyl-(n)Lc4Cer (m/z 1407.8), and the six acidic GSLs were deduced to be GM3 (m/z 1151.7), GM2 (m/z 1354.8), GM1 (m/z 1516.8), GD3, (m/z 720.9) GD2 (m/z 822.4), and GD1a/GD1b (m/z 903.5). There were no significant differences between the EB outgrowths differentiated from MRC-iPSCs and UtE-iPSCs. Moreover, these GSLs in EB outgrowths were similar to those found in NSCs. The analysis of GSL immediately after EB formation was also carried out. The detected GSLs were a mixture of the GSLs observed in hiPSCs and EB outgrowth.

### Immunocytochemical and flow cytometric analysis of parental cells, hiPSCs, NSCs, and EB outgrowth

**Figure 3:**
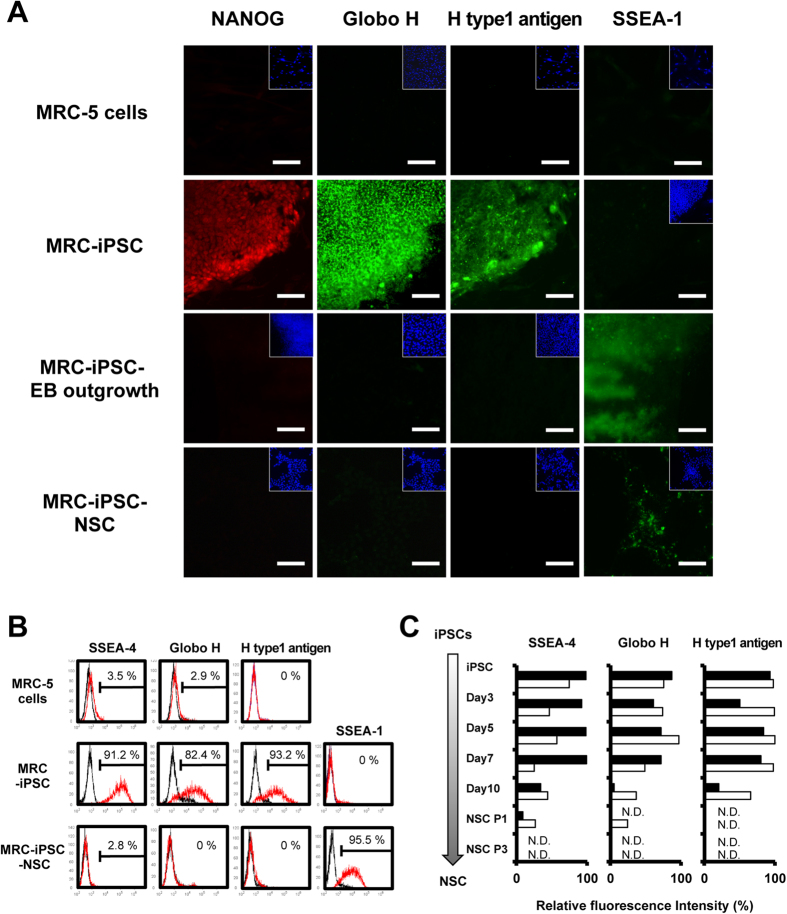
GSL expression in iPSCs, their parental cells and iPSC-NSCs. (**A**) Immunocytochemical analyses of NANOG, Globo H, H type1 antigen, and SSEA-1 in MRC-5, MRC-iPSCs, MRC-iPSC-EB outgrowth, and MRC-iPSC-NSCs. Inserted images are nuclear staining by DAPI. Scale bar is 100 μm. (**B**) Flow cytometric analyses of GSLs in MRC-5, MRC-iPSCs, and MRC-iPSC-NSCs. SSEA-4, Globo H, H type1 antigen and SSEA-1 were analyzed using GSL-specific antibodies versus the isotype controls. Cells stained with specific antibodies are shown in red, and isotype antibodies are shown in black. (**C**) Time course analyses by flow cytometry of SSEA-4, Globo H, and H type1 antigen during the process of differentiation from iPSCs to NSCs. Black columns represent MRC-iPSCs and white columns, UtE-iPSCs. N.D. is “not detected”.

The results of GSL analysis in parental cells, hiPSCs, NSCs, and EB outgrowth are summarized in Table 2. In addition to Gb5Cer and sialyl-Gb5Cer corresponding to SSEA-3 and SSEA-4, which are markers for undifferentiated hiPSCs, fucosyl-Gb5Cer and IV fucosyl-(n)Lc4Cer corresponding to Globo H and H type 1 antigen, were specifically expressed only in undifferentiated hiPSCs. To confirm the GSL analysis, immunocytochemical analysis and flow cytometric analysis using antibodies against SSEA-4, Globo H, H type1 antigen, and SSEA-1 were carried out. Immunocytochemical analyses showed that MRC-iPSCs were positive for Globo H and H type1 antigen (Fig. 3A), and NSCs and EB-outgrowth were positive for SSEA-1. Flow cytometric analyses also revealed that the MRC-iPSCs were positive for Globo H, H type1 antigen, and SSEA-3, and were negative for SSEA-1. NSCs were positive for SSEA-1 (Fig. 3B). Similar results were also obtained for iPSCs, NSC, and EB-outgrowth derived from UtE cells as parent cells (Supplemental Figure S3B, S4). These results suggest that fucosyl-(n)Lc4Cer detected in iPSCs was IV fucosyl-Lc4Cer corresponding to H type 1 antigen, and fucosyl-(n) Lc4Cer detected in NSCs and EB outgrowth was III fucosyl-nLc4Cer corresponding to SSEA-1. Time-dependent expression of SSEA-4, Globo H and H type 1 antigen were also measured during the differentiation process of iPSCs to NSCs. These antigens were no longer expressed after cultivation of the cells in the NSC differentiation medium for 6 days followed by cultivation in NSC maintenance medium for 3 days (Fig. 3C).

### Determination of ABO genotyping

**Figure 4:**
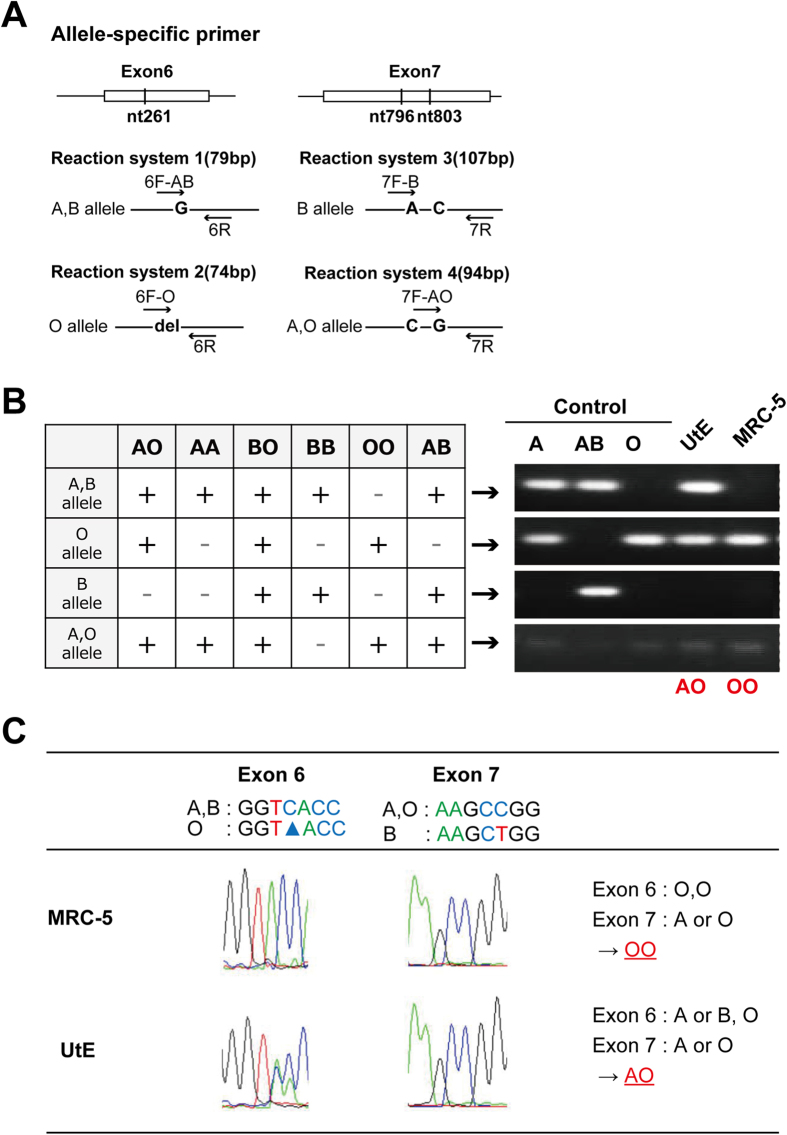
Determination of ABO genotypes. (**A**) Allele-specific primers for the ABO transferase gene. (**B**) ABO genotyping for MRC and UtE cells. Each RT-PCR gel was electrophoresed at the same time. The gels were cropped to show the corresponding bands clearly. (**C**) Sequence analysis of the genes for ABO system transferase.

Because both MRC-iPSCs and UtE-iPSCs express H antigen (blood type O), ABO genotyping of both cell types was carried out by two different methods^20^,^21^. Both the allele-specific primer method and sequencing of the ABO transferase gene revealed that MRC-5 cells and UtE cells carried genotypes for blood types O and A, respectively (Fig. 4). These results imply that iPSCs express H antigen (blood type O) when in the undifferentiated state, irrespective of blood group antigen genotype.

### Genetic alteration of glycan biosynthesis during iPSC generation

**Figure 5:**
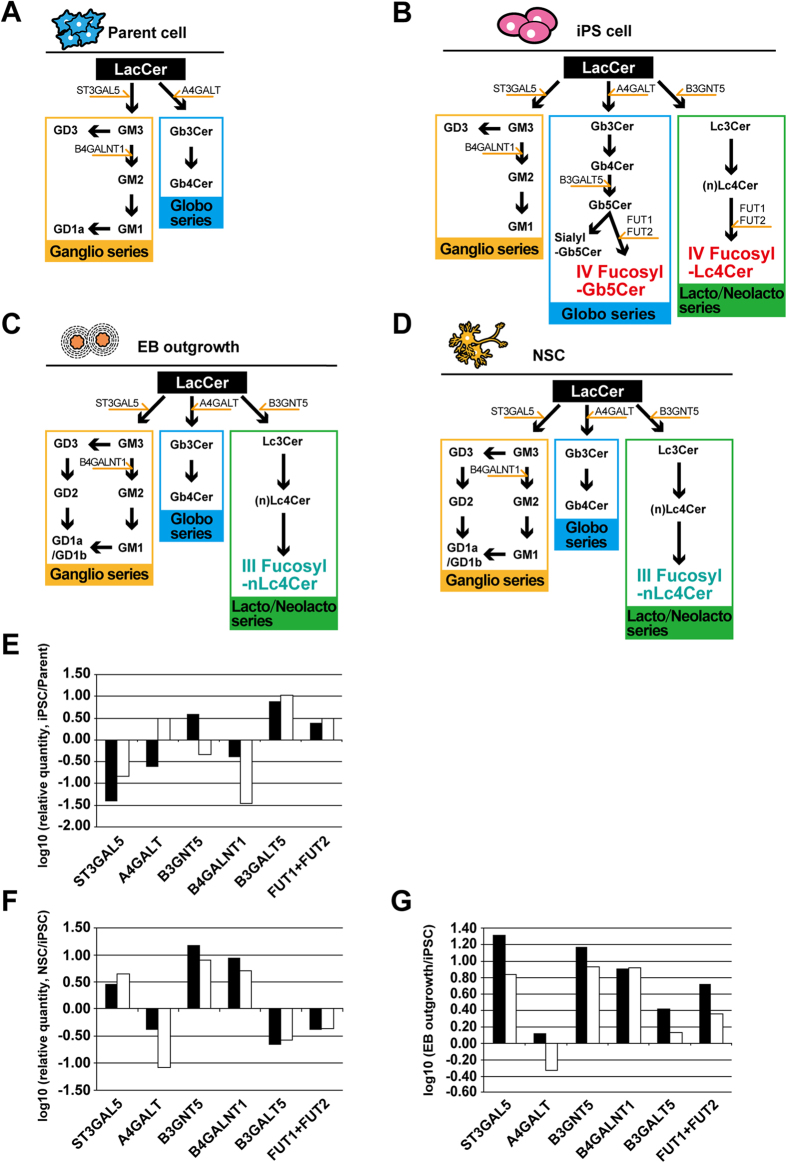
Biosynthetic pathways of GSLs and expression of glycosyltransferase genes. (**A**–**D**) Biosynthetic pathways of GSLs in parental cells (**A**), iPSCs (**B**), EB-outgrowth (**C**), and NSCs (**D**). (**E**) Relative quantity of glycosyltransferase genes expressed in MRC-iPSCs and UtE-iPSCs compared to their parental cells. The ratios of MRC-iPSC(#25)/MRC-5 (black columns) and UtE-iPSC(B05)/UtE (white columns) were plotted on a log scale bar graph. (**F**,**G**) Relative amount of glycosyltransferase genes expressed in iPSC-NSCs and iPSC-EB outgrowth against iPSCs. The ratios of iPSC-NSC/iPSCs (**F**) and iPSC-EB outgrowth/iPSCs (**G**) were plotted on a log scale bar graph. The black columns mean that the parental cells are MRC-5 cells, and the white columns mean that the parental cells are UtE cells. Akihiro Umezawa drew these figures by himself.

The biosynthetic pathways for the GSLs observed in these studies are shown in Fig. 5A–D and Table 2. The major glycosyltransferase genes related to synthesis of the GSLs are also indicated (Fig. 5A–D). In Table 2, several GSLs were not detected in the present study. However, we could not exclude the possibility of the presence of the GSLs. To determine whether the proposed GSL synthetic pathways were actually functional in parental cells and hiPSCs, quantitative RT-PCR was carried out for the glycosyltransferase genes ST3GAL5, A4GALT, B3GNT5, B4GALNT1, B3GALT5, FUT1 and FUT2. The copy numbers of those genes ranged from 10^3^–10^6^/1μg RNA. The relative values of iPS/parent cells were evaluated for each gene (Fig. 5E). Expression of ST3GAL5 and B4GALNT1, which function in the biosynthesis of ganglio-series GM3 and GM2, respectively, were significantly decreased after iPSC generation. The expression of B3GNT5, responsible for the biosynthesis of Lc3Cer, was up-regulated in MRC-iPSCs, but was down-regulated in UtE-iPSCs. In contrast, A4GALT, which functions in globo-series GSL biosynthesis, was down-regulated in MRC-iPSCs, but in UtE-iPSCs, it was up-regulated. Based on these results, the increase in globo- and lacto/neolacto-series GSLs in hiPSCs was a result of the decreased expression of the GM3 synthase gene ST3GAL5. Though the predominant glycan synthetic pathway in the parental cells is for ganglio-series GSLs, the predominant pathways in hiPSCs switched to the globo- and lacto-series GSLs, due to the large decrease in GM3 synthase. Additionally, FUT1 and FUT2, which are related to biosynthesis of Globo H, H type1 antigen and other H antigens, were upregulated in hiPSCs. Therefore, we conclude that the expression of fucosyl-Gb5Cer and IV fucosyl-(n)Lc4Cer in hiPSCs were influenced by both the increase of FUT1/FUT2 and the increase in globo- and lacto/neolacto-series GSL biosynthesis. Accordingly, three kinds of MRC-iPSCs and two kinds of UtE-iPSCs were prepared in order to examine the expression of these glycosyltransferase genes, and the results were similar for all five iPSCs (Supplemental Figure S5).

### Glycan biosynthesis-related genes in iPSC-derived NSCs and EB outgrowth

To investigate expression of glycan biosynthesis-related genes during differentiation from iPSCs to NSCs and during EB outgrowth, quantitative RT-PCR for the genes ST3GAL5, A4GALT, B3GNT5, B4GALNT1, B3GALT5, FUT1 and FUT2 was carried out in NSCs and EB-outgrowth (Fig. 5F,G). ST3GAL5 and B4GALNT1, responsible for biosynthesis of ganglio-series, increased in NSCs and EB outgrowth that had been differentiated from MRC-iPSCs and UtE-iPSCs. In contrast, A4GALT, involved in biosynthetic pathway of globo-series GSLs such as Gb5Cer, sialyl-Gb5Cer, and fucosyl-Gb5Cer, was down-regulated. B3GNT5, the Lc3Cer synthase gene, showed an increase in expression during differentiation of hiPSCs to EB growth and NSC, FUT1/FUT2 expression increased in EB outgrowth, but not in NSCs.

## Discussion

GSLs presented on the surface of mammalian cells participate many important biological functions, but analysis of the expression of GSLs in hESCs has only been reported recently^16^,^17^,^18^. Though hESCs and hiPSCs are considered to be identical in many aspects including cell surface markers, expression of GSLs in hiPSCs has not been reported. In the present study, glycolipid dynamics during generation and differentiation of hiPSCs was investigated using LC-MS. Dynamic gene expression and epigenetic changes continues for more than 50 weeks after iPSC generation^22^,^23^. In our study, global images of GSLs determined by LC-MS clearly showed drastic alterations immediately after iPS generation (Table 2). Several GSLs were not detected in the present study. In some cases, the GSLs might not be detected because the measurement was beyond the limits of sensitivity. The lipid extracts without alkaline hydrolysis were also analyzed by LC-MS to eliminate possibility of GSL hydrolysis. However, no differences in the variety of GSLs between the lipid extracts with or without alkaline hydrolysis were observed in the present study. We could not determine structure of some GSLs due to lack of, or low levels of GSLs (“not detected” in Table 2). The GSL panel in Table 2 indicates the distinctive expression of globo-series and lacto/neolacto-series in undifferentiated iPSCs, compared to parental somatic cells and differentiated forms of hiPSCs. Consistent correlation between the expression levels of GSLs and the corresponding glycosyltransferases strongly suggests that regulation of glycosyltransferase genes are the direct cause of glycolipid dynamics during generation of iPSCs. The significant down-regulation of ST3GAL5 and B4GALNT1 correlated with the biosynthesis of ganglio-series GSLs and influenced the increase of globo and lacto/neolacto-series GSLs. Up-regulation of A4GALT in UtE-iPSCs and B3GNT5 in MRC-iPSCs also resulted in an increase of globo and lacto/neolacto-series GSLs. Gaglio-, globo-, and lacto/neolacto-series GSLs were synthesized from lactosylceramide as a precursor. Since GM3, Gb3Cer, and Lc3Cer are competitively biosynthesized in cells, down-regulation of GM3 biosynthesis would induce the up-regulation of the biosynthesis of Gb3Cer and Lc3Cer.

SSEA-3 and SSEA-4 are markers of undifferentiated hiPSCs. In our study, the structures of GSLs corresponding to SSEA-3 and SSEA-4 were deduced to be Gb5Cer and sialyl-Gb5Cer, respectively, by MS/MS analysis. In addition to SSEA-3 and SSEA-4, Globo H was also detected by immunocytochemical analysis. The structure of Globo H was deduced to be fucosyl Gb5Cer, which is synthesized by α1-2 fucosyltransferase (*FUT1*/*FUT2*). The expression of FUT1/FUT2 in hiPSCs showed a pronounced increase over their expression in the parental cells, and the biosynthesis of fucosyl Gb5Cer correlated with the up-regulation of FUT1/FUT2. The same genes also catalyze the biosynthesis of H type1 antigen. Indeed, IV fucosyl-(n)Lc4Cer in hiPSCs was detected by LC-MS, and the hiPSCs were positive for immunostaining with an anti-H type 1 antibody.

H antigen, the blood type O determinant, was detected in iPSCs. Although the parental cells, MRC5 and UtE5 cells, had genotypes of blood type O (OO) and type A (AO), respectively, both hiPSCs phenotypically expressed only H antigen. Cell surface ABO blood types are critical for transplantation of organs such as liver and kidney^24^. The expression of H antigen in hiPSCs, regardless of the parental genotype, has important ramifications for the use of hiPSCs in transplantation.

The GSLs in hESCs have been identified as globo- and lacto/neolacto-series GSLs including Gb4Cer, Gb5Cer, sialyl-Gb5Cer, fucosyl-Gb5Cer, Lc4Cer, and IV fucosyl-Lc4Cer, and using flow cytometry and mass spectrometry^16^. More recently, Barone et al. carried out the further identification of 18 kinds of globo- and lacto/neolacto-series GSLs including (n)Lc4Cer, H type1 pentaosylceramide and H type2 pentaosylceramide by mass spectrometry and proton NMR^18^. The GSLs observed in our study using hiPSCs have been also detected in hESCs. These results indicate that hiPSCs and hESCs display similar GSL synthetic pathways.

Recently, an antibody against SSEA-5 (H type 1 glycan) was proposed to detect hiPSCs and ESCs and remove teratoma-forming cells^25^. However, it has not been determined whether the carbohydrate antigen is glycolipid or glycoprotein in hiPSCs. More recently, an O-glycan comprising an H type 3 antigen structure in hiPSCs was identified using a high-throughput antibody-overlay lectin microarray^9^. These papers and our present study suggest that multiple structures of H antigen exist on the cell surface of hiPSCs.

Based on the GSL analysis for hiPSCs, fucosyl-Gb5Cer and IV fucosyl-(n)Lc5Cer appear to be good candidates as novel markers for validation and identification of hiPSCs. To determine the specificity of the two GSLs detected in undifferentiated hiPSCs, the GSLs in NSCs and EB outgrowth that were differentated from hiPSCs were also identified by LC-MS. Fucosyl-Gb5Cer and IV fucosyl-(n)Lc4Cer, as well as Gb5Cer and sialyl-Gb5Cer, were not detected in NSCs and EB outgrowth. The analysis of GSL-related glycosyltransferase genes suggest that the disappearance of Gb5Cer, sialyl-Gb5Cer, and fucosyl-Gb5Cer were caused by the increase of ST3GAL5 and B4GALNT5 activity, which are related to the biosynthesis of ganglio-series GSLs; and the decrease of A4GALT, which is related to the biosynthesis of globo-series GSLs.

The switching of the core structures of GSLs was observed for differentiation of hiPSCs as well as hESCs. Alteration of GSLs during differentiation of ESCs to EB outgrowth has also been reported^16^. It was also demonstrated that the core structures of GSLs in EB outgrowth switched from globo- and lacto/neolacto- to ganglio-series, and GM3 synthase (ST3GAL5) activity increased significantly. Furthermore, the switching of the core structures of GSLs from globo- and lacto/neolacto- to ganglio-series has been observed in mouse embryonic development as well as human ESC differentiation^5^. However, the expression of SSEA-1 in EB outgrowth, neural progenitor cells and definitive endoderm was not reported in this previous study. We found that SSEA-1 known as NSC marker was detected in NSCs and EB outgrowth by immunocytochemical analysis. The GSL structures corresponding to H type 1 antigen and SSEA-1 are isomers of fucosyl-(n)Lc4Cer. Though the sequences were determined by the MS/MS analyses, the isomers such as lacto- and neolacto-series could not be determined and the coexistence of the isomers could not be excluded by the MS/MS analyses. The proposed structures of the GSLs were deduced by the immunocytochemical analysis.

Interestingly, the cell type-specific major fatty acid length was detected: “C16:0 and C18:0” in iPSCs, and “C16:0 and C24:0” in parental cells, NSC, and EB cells. This difference in major fatty acid length may be attributed to the alteration in the biosynthesis of ceramide during the process of reprograming and differentiation.

In conclusion, we observed the GSL dynamics that occurs during reprogramming and differentiation of hiPSCs. The predominant GSL biosynthesis in undifferentiated hiPSCs consisted of globo- and lacto/neolacto-series GSLs including SSEA-3, SSEA-4, Globo H, and H type antigen, while those in parental somatic cells, NSC, and EB outgrowth were comprised of ganglio-series GSLs. Switching of GSLs from ganglio- to globo- and lacto/neolacto-series in hiPSCs may contribute to maintenance of the undifferentiated state and pluripotency. However, the roles of SSEA-3 (Gb5Cer), SSEA-4 (sialyl-Gb5Cer), Globo H (fusosyl-Gb5Cer), and H type1 antigen (IV fusosyl-Lc4Cer) in the functions of hiPSCs have not been clarified. It has been reported that SSEA-3 and Globo H were expressed in cancer stem cells^26^, and that Globo H was expressed in breast cancer^27^. Therefore, analysis of the roles of globo- and lacto/neolacto-series GSLs in hiPSCs may help elucidate the mechanisms of generation and differentiation of cancer cells.

## Methods

### Cell Culture

hiPSCs were maintained with irradiated MEFs as previously described^8^,^22^,^23^,^28^. In this study, MRCiPS#16 (also called Fetch/NIHS0604 in other literature), MRCiPS#25 (Tic/JCRB1331), MRCiPS#40 (Skipper/NIHS0600), UtE-iPS-A07 (UtEiPS-07/QuarterBack), and UtE-iPS-B05 (UtEiPS-11/SplitEnd) were used for carbohydrate analysis. The MRC-iPSCs and UtE-iPSCs were generated from parental cells, MRC-5 cells and uterine endometrial cells (UtE, specimen number 1104). Differentiation of iPSCs into NSC was carried out according to the standard protocol^29^. iPSCs were cultivated in medium for inducing differentiation into NSC for 7 days followed by NSC maintenance medium for 3 days (Supplemental Figure S2A, Supplemental Table S3A). Differentiation of iPSCs to EB outgrowth was carried out as follows: iPSCs were treated with 300PU/ml dispase II (Invitrogen) at 37 °C for 3 min. After removal of irrMEF (irradiated mouse embryonic fibroblast), colonies of iPSCs were collected by pipetting with EB medium (Supplemental Table S3B). The iPSCs were washed with EB medium three times. The cells were suspended with EB medium, and the cells were seeded onto Lipidure^®^ Coat Dishes A-90 (NOF Co.), and incubated in 5% CO_2_-95% air at 30 ˚C for 4 days. Thereafter, the cells were cultured in D-MEM/F-12 (Gibco) supplemented with 10% FBS (Gibco) and 1% penicillin/ streptomycin (Gibco) for 10 days. At 11days after the culture in D-MEM/F-12 (Gibco) supplemented with 10% FBS and 1% penicillin/ streptomycin, EB out growth was formed, and tridermic cells were observed.

### Immunocytochemical analysis

Cells were fixed with 4% paraformaldehyde in PBS at 4 °C for 10 min. After washing with PBS, the cells were pre-hybridized with blocking buffer (10% goat serum in PBS) for 30 min at room temperature and then reacted with the primary antibodies in blocking buffer at 4 °C for 12 h. After washing with PBS, the cells were reacted with the secondary antibodies in blocking buffer for 30 min at room temperature. Primary and secondary antibodies are summarized in Supplemental Table S4A–C. The cells were counterstained with DAPI and mounted.

### cDNA standards

Genes for A4GALT, ST3GAL5, B3GNT5, B4GALNT1, B3GALT5, FUT1, and FUT2 were amplified with primers shown in Supplemental Table S5A from cDNA prepared from MRC-5 cells (A4GALT and ST3GAL5), UtE cells (B4GALNT1), MRC5-iPSCs (B3GALT5), UtE-iPSCs (FUT1 and FUT2), and UET13 cells (B3GNT5) by PCR using KOD plus ver.2 (TOYOBO) or KOD plus neo (TOYOBO) and cloned into pENTR11 (Invitrogen) pGEM-T easy vector (Promega) and pcDNA3 (Invitrogen). cDNA plasmid concentrations were measured by optical density spectrometry and corresponding copy number was calculated using the following equation: 1 ng of 1000 bp DNA = 9.1 × 10^8^ molecules. Serial dilutions from the cDNA plasmid were used as standard curves, each containing a known amount of input copy number.

### Quantitative RT-PCR

RNA was extracted from cells using the RNeasy Plus Mini kit (Qiagen). An aliquot of total RNA was reverse transcribed using an oligo(dT) primer. For the thermal cycle reactions, the cDNA template was amplified (ABI PRISM 7900HT Sequence Detection System) with primers shown in Supplemental Table S5B using the Platinum Quantitative PCR SuperMix-UDG with ROX (11743-100, Invitrogen) under the following reaction conditions: 40 cycles of PCR (95 °C for 15 s and 60 °C for 1 min) after an initial denaturation (95 °C for 2 min). Fluorescence was monitored during every PCR cycle at the annealing step. The authenticity and size of the PCR products were confirmed using a melting curve analysis (with software provided by Applied Biosystems) and gel electrophoresis analysis.

### LC-MS/MS

Lipids were extracted from the cell pellets with chloroform/methanol (2:1, v/v) and chloroform/isopropanol/water (7:11:2, v/v) in a sonicating water bath. Total extracts were mixes and evaporated to dryness. Alkaline hydrolysis was performed to remove phospholipids. The lipid extracts without alkaline hydrolysis were also prepared. The lipids extracted from the cells were dissolved with chloroform/methanol (2:1, v/v) and 2 M KOH was added. After incubation at 37 °C for 3 hours, supernatants were collected and evaporated to dryness. The extracts were desalted using a SepPak C18 column (Waters) and analyzed by LC-MS. LC-MS employed in this study was electrospray ionization (ESI)/an ion trap mass spectrometer (HCT-Ultra 11S, Bruker Daltonics, Billerica, MA, USA) equipped with the ADVANCE UHPLC (AMR, Japan). The glycolipids were loaded onto a silica column (0.2 × 150 mm, Imtakt, Japan) equilibrated with solvent A (chloroform/methanol/water (C/M/W) containing 2 M ammonium acetate buffer, pH 5.5 (89/10/1, v/v/v)), and were eluted with a 0–100% linear gradient of solvent B (M/W containing 0.2 M ammonium acetate buffer, pH 5.5 (9/1, v/v)) in 60 min at a flow rate of 2000 nl/min. The ESI parameters were as follows: nebulizer, 7.0 psi; dry gas (N_2_), 4.0 l/min; dry temperature, 300°C; capillary, −4 kV for negative ion mode. The mass recorded in survey acquisitions ranged from 150 through 1,800 m/z. NSC and hiPSCs were detected by multiple reaction monitoring mode for (iPSCs, NSCs, and EB outgrowth) and auto MS mode (parental cells).

### ABO genotyping

Genomic DNA was isolated using the DNeasy kit (Qiagen) according to the manufacturer’s protocol. ABO blood types were determined by the PCR-APLP method using the allele-specific primers^20^. In addition, exons 6 and 7 of the ABO transferase gene was sequenced after amplification by genomic PCR using specific primers^21^.

### Flow cytometry

Cells were stained for 1 h at 4 °C with primary antibodies and immunofluorescent secondary antibodies. The cells were then analyzed on a Cytomics FC 500 (Beckman Coulter, Inc.) and the data were analyzed with the FlowJo Ver. 7 (Tree Star, Inc.). Antibodies against human Globo H (ALX-804-550C050, Enzo Life Sciences, Inc.) and H antigen (anti-blood group H1 (O) antigen antibody:ab3355, abcam) were adopted as primary antibodies. Alexa Fluor 488 goat anti-mouse IgM (μ chain, A21042, Invitrogen) and Alexa Fluor 488 F(ab’)2 fragment goat anti-mouse IgG (H + L) (A11017, Invitrogen) were used as secondary antibodies when the primary antibodies were to Globo H and H antigen, respectively. Fluorescent-conjugated antibodies (555401, BD Pharmingen and FAB1435F, R&D) were used for the analyses of SSEA-1 and SSEA-4, respectively. Isotype antibodies were used as control (Supplemental Table S5C).

## Additional Information

**How to cite this article**: Ojima, T. *et al.* Glycolipid dynamics in generation and differentiation of induced pluripotent stem cells. *Sci. Rep.* **5**, 14988; doi: 10.1038/srep14988 (2015).

## Supplementary Material

Supplementary Information

## Figures and Tables

**Table 1:** GSLs detected in parental cells.

Proposed GSLs	Cer	Ion Type	Calculated Mass(m/z)	Observed Mass (m/z)	
				MRC-5	UtE
GlcCer	d18:1/24:0	[M + Cl]^–^	846.7	848.4	847.4
	d18:1/16:0	[M + Cl]^–^	735.1	N.D.	N.D.
LacCer	d18:1/24:0	[M + Cl]^–^	1008.7	1008.4	1008.4
	d18:1/16:0	[M + Cl]^–^	896.6	896.4	N.D.
Gb3Cer	d18:1/24:0	[M + Cl]^–^	1170.8	1170.4	1170.9
	d18:1/16:0	[M + Cl]^–^	1058.6	1059.1	1058.9
Gb4Cer	d18:1/24:0	[M + Cl]^–^	1373.8	1372	N.D.
	d18:1/16:0	[M + Cl]^–^	1261.7	N.D.	1261.9
GM3	d18:1/24:0	[M-H]^–^	1263.8	1263.4	1263.3
	d18:1/16:0	[M-H]^–^	1151.7	1152.1	1152.4
GM2	d18:1/24:0	[M-H]^–^	1466.9	1466.9	1466.6
	d18:1/16:0	[M-H]^–^	1354.8	1355.4	1355.1
GM1	d18:1/24:0	[M-H]^–^	1629	N.D.	1628.4
	d18:1/16:0	[M-H]^–^	1516.8	1517.1	1517.7
GD3	d18:1/24:0	[M-2H]^2–^	777	777	776.8
	d18:1/16:0	[M-2H]^2–^	720.9	720.9	720.9
GD1a	d18:1/24:0	[M-2H]^2–^	959.5	959.2	959.2
	d18:1/16:0	[M-2H]^2–^	903.5	904.9	903.8

N.D. means “not detected”.

**Table 2:** GSLs detected in parental cells, iPSCs, EB-outgrowth, and neural stem cells.

Proposed GSLs	Parental cells		iPSC		EB-outgrowth		NSC	
	MRC-5	UtE	MRC-5	UtE	MRC-5	UtE	MRC-5	UtE
GlcCer	+	N.D.	+	+	+	+	+	+
LacCer	N.D.	+	+	+	+	+	+	+
Gb3Cer	+	+	+	+	+	+	+	+
Gb4Cer	+	+	+	+	+	+	+	+
Gb5Cer	N.D.	N.D.	+	+	N.D.	N.D.	N.D.	N.D.
Fucosyl-Gb5Cer	N.D.	N.D.	+	+	N.D.	N.D.	N.D.	N.D.
HexNAc+ Gb5Cer	N.D.	N.D.	N.D.	N.D.	N.D.	N.D.	N.D.	N.D.
Sialyl-Gb5Cer	N.D.	N.D.	+	+	N.D.	N.D.	N.D.	N.D.
Lc3Cer	N.D.	N.D.	+	+	+	+	+	+
(n)Lc4Cer	N.D.	N.D.	+	+	+	+	+	+
IV Fucosyl-(n)Lc4Cer	N.D.	N.D.	+	+	N.D.	N.D.	N.D.	N.D.
III Fucosyl-(n)Lc4Cer	N.D.	N.D.	N.D.	N.D.	+	+	+	+
GM3	+	+	+	+	+	+	+	+
GM2	+	+	+	+	+	+	+	+
GM1	+	+	+	+	+	+	+	+
GD3	+	+	+	+	+	+	+	+
GD2	N.D.	N.D.	+	N.D.	+	+	+	+
GD1a/GD1b	+	+	N.D.	N.D.	+	+	+	+

N.D. means “not detected”.
