# Supplementary material for: Glycolipid dynamics in generation and differentiation of induced pluripotent stem cells
Source: Sci Rep. 2015 Oct 19;5:14988. doi: 10.1038/srep14988 (PMC4609952; doi:10.1038/srep14988)
Supplement: Supplementary Information [file srep14988-s1.pdf]

Supplementary Information

Glycolipid dynamics in generation and differentiation of induced pluripotent stem cells

Takuma Ojima, Eri Shibata, Shiho Saito, Masashi Toyoda, Hideki Nakajima, Mayu Yamazaki-Inoue, Yoshitaka Miyagawa, Nobutaka Kiyokawa, Jun-ichiro Fujimoto, Toshinori Sato, and Akihiro Umezawa

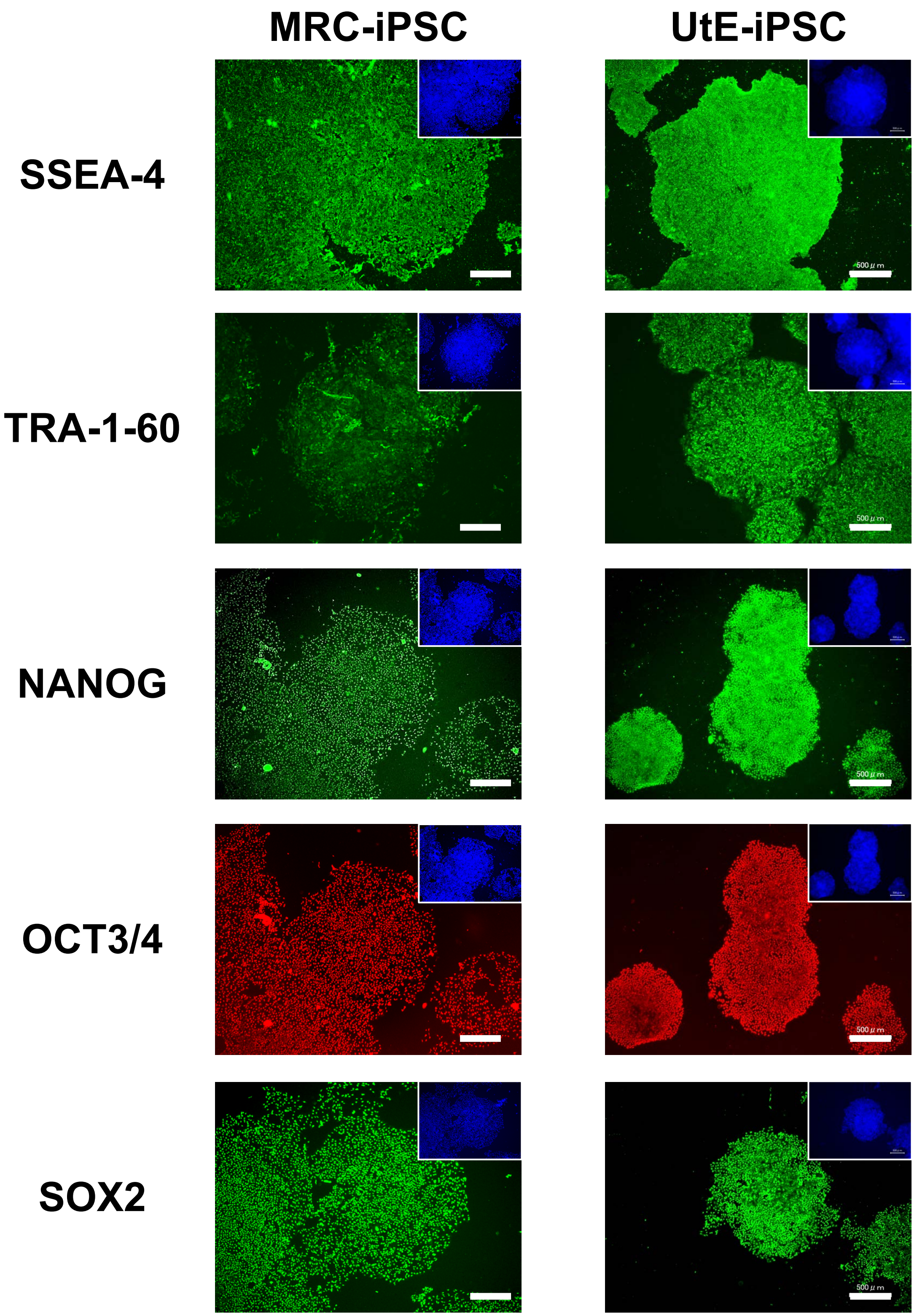

Figure S1. Immunocytochemistry of SSEA-4, TRA-1-60, NANOG, OCT3/4, SOX2 in MRC-iPSCs and UtE-iPSCs
Inserted images are nuclear staining by DAPI. Bar is 100 μm.

Supplemental Figure S1

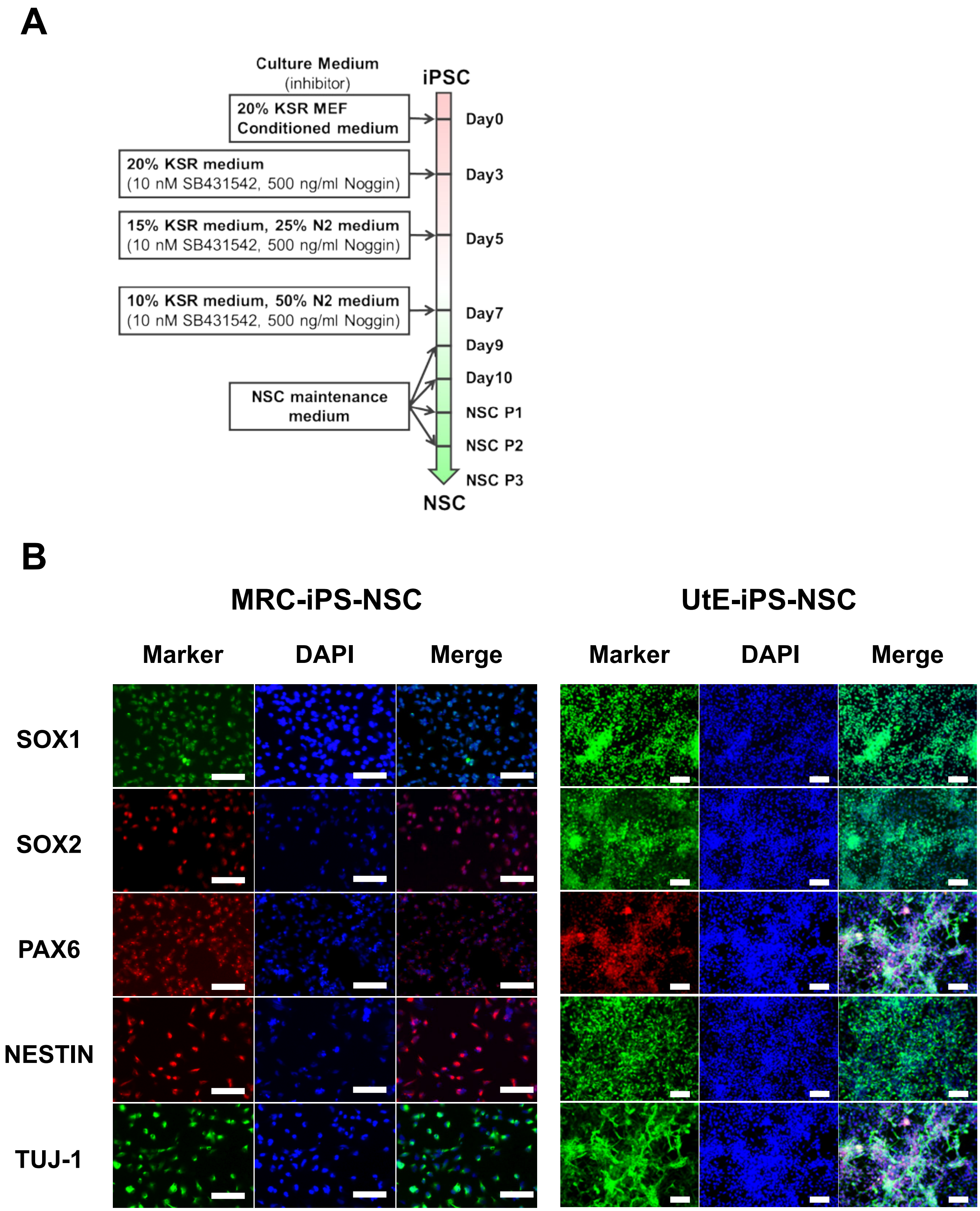

Supplemental Figure S2. Immunocytochemistry in iPSC-NSCs
A. Experimental procedure for neural differentiation of iPSCs.
B. Immunocytochemical analyses of SOX1, SOX2, PAX6, NESTIN, and TUJ-1 in MRC-iPSC-NSCs and UtE-iPSC-NSCs. Inserted images are nuclear staining by DAPI. Bar is 100 μm.

Supplemental Figure S2

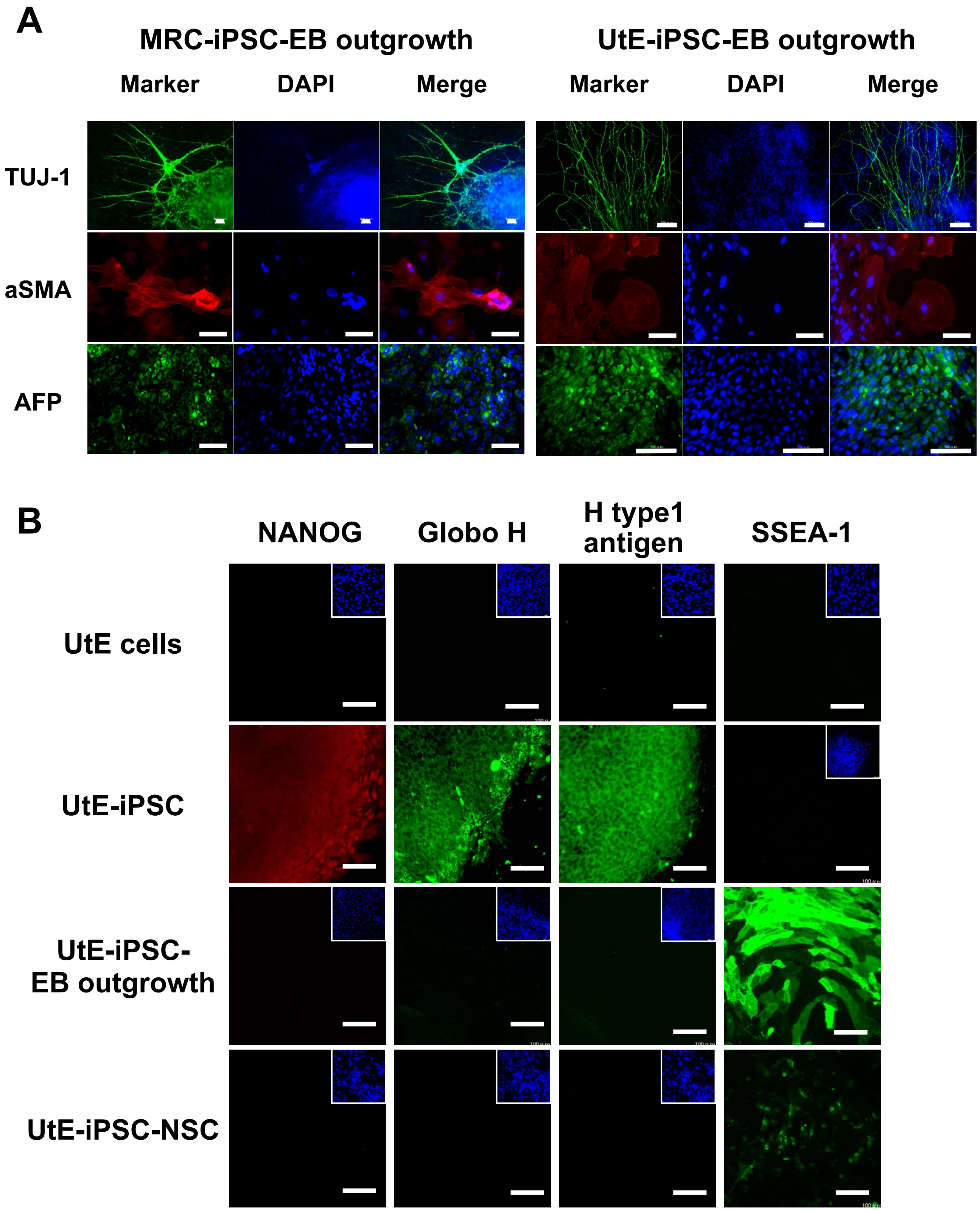

Supplemental Figure S3. Immunocytochemistry of iPSCs after embryoid body (EB) outgrowth

A. Immunocytochemical analyses of TUJ-1, α-smooth muscle actin (aSMA), and α-fetoprotein (AFP) in MRC-iPSC-EB outgrowth and UtE-iPSC-EB outgrowth. Inserted images are nuclear staining by DAPI. Bar is 100 μm. **B**. Immunocytochemical analysis of NANOG, GloboH, H type1 antigen SSEA-1 in UtE, UtE-iPSCs, UtE-iPSC-NSC, and UtE-iPSC-EB outgrowth. Inserted images are nuclear staining by DAPI. Bar is 100 μm.

Supplemental Figure S3

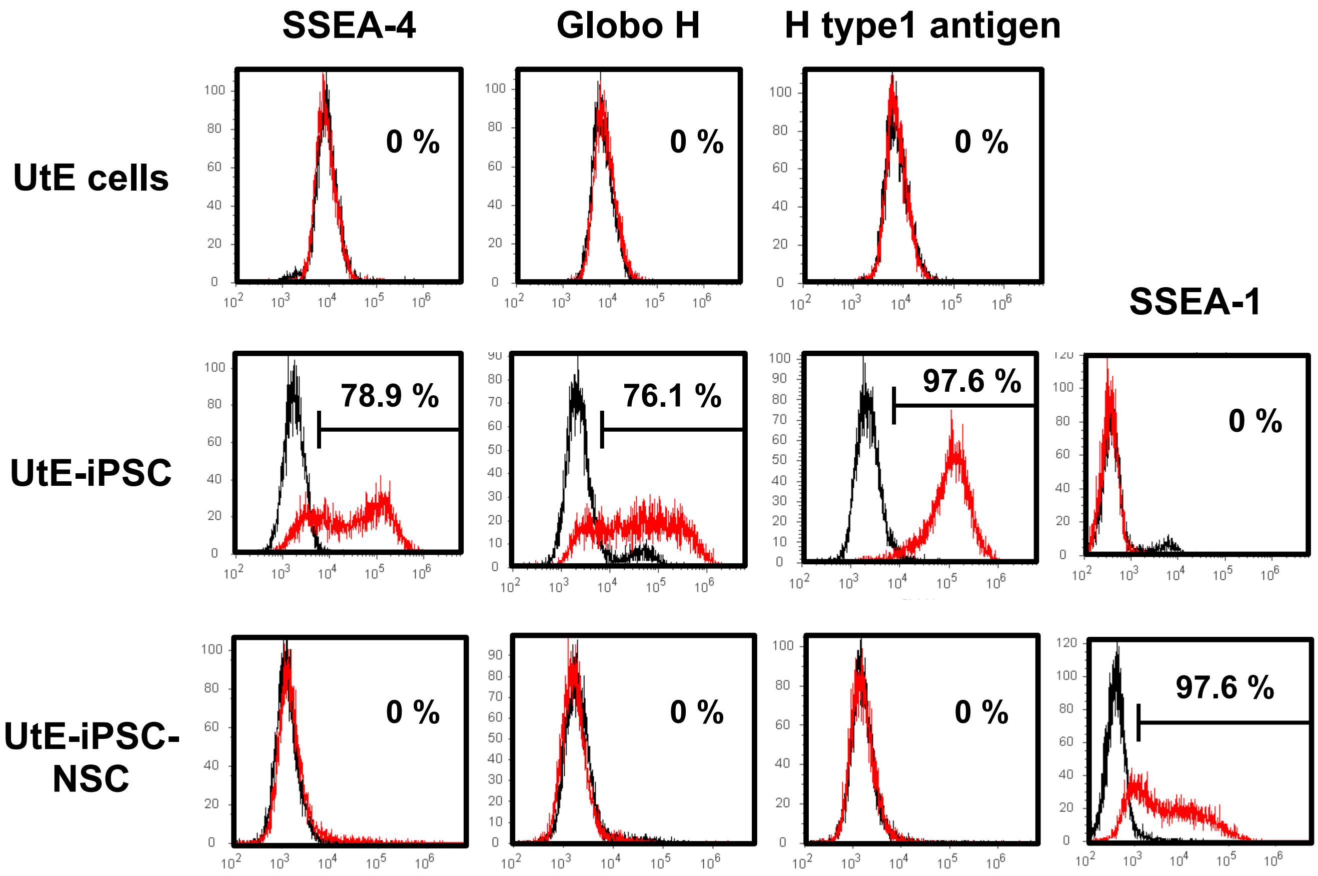

Supplemental Figure S4. Flow cytometric analyses of GSLs in UtE parental cells, UtE-iPSCs, and UtE-iPSC-NSCs

The indicated cells were analyzed for SSEA-4, Globo H, H type1 antigen and SSEA-11 with GSL-specific antibodies versus the isotype controls. Cells stained with the specific antibodies are shown in red, and isotype antibodies are shown in black.

Supplemental Figure S4

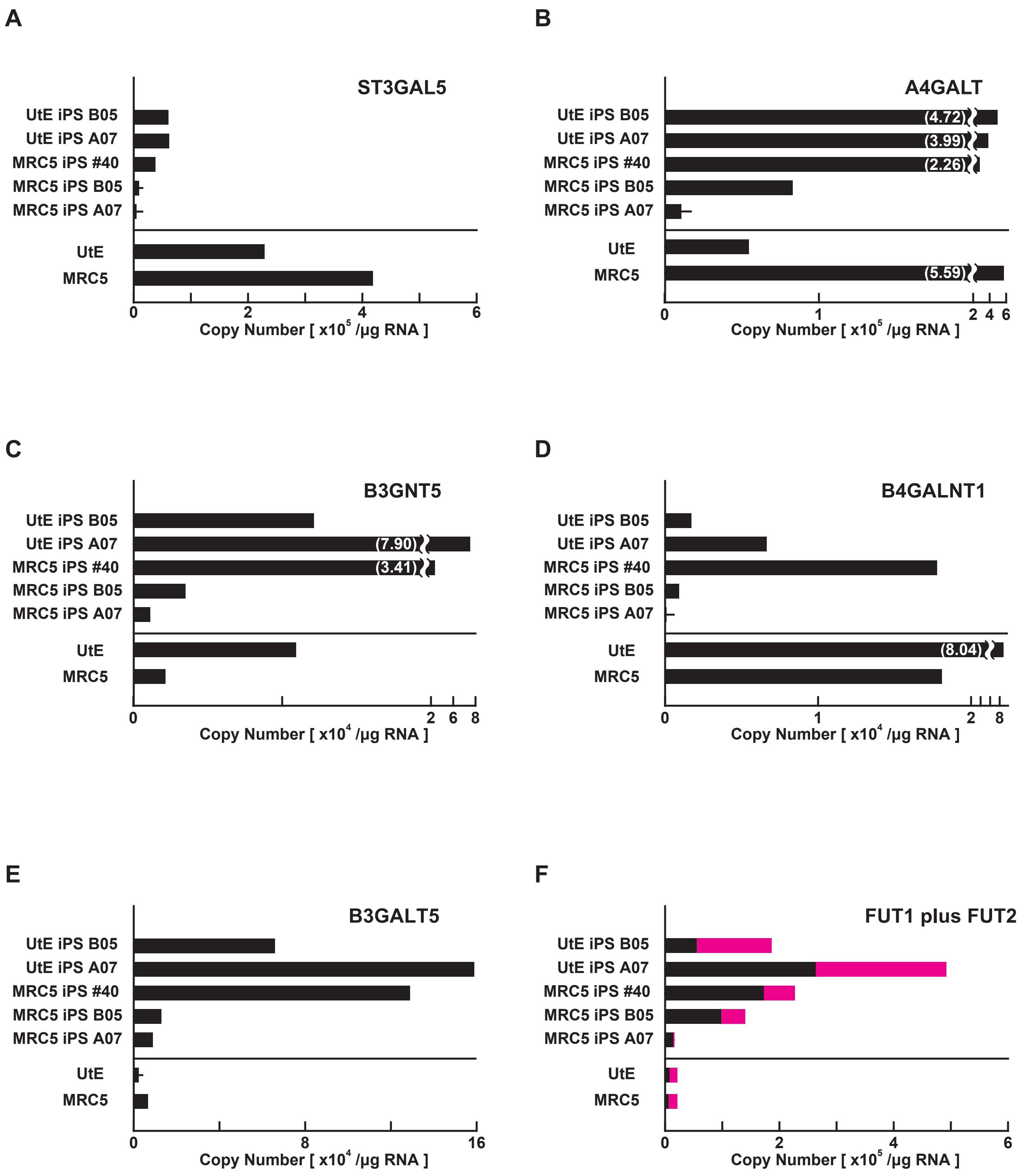

Supplemental Figure S5. Expression of glycosyl transferase genes in iPS cells and their parental cells

Gene expression levels (A:ST3GAL5, B:A4GALT, C:B3GNT5, D:B4GALNT1, E:B3GALT5, F:FUT1 and FUT2) are shown as copy number of each gene per one microgram total RNA. RNAs were obtained from UtEiPS-B05, UtEiPS-A07, MRCiPS#40, MRCiPS#25, MRCiPS#16, parental uterine endometrial cells (UtE), and MRC-5 cells (from top to bottom).

Supplemental Figure S5

Supplemental Table S1A. List of GSL structures in MRC-5 cells by LC-MS or MS/MS

Proposed GSLs	Fragments
GlcCer	810.9 ([M-H]$^-$), 648.6 ([Y_0-H]$^-$)
LacCer	972.8 ([M-H]$^-$), 810.8 ([Y_1-H]$^-$), 648.9 ([Y_0-H]$^-$)
Gb3Cer	1134.9 ([M-H]$^-$), 972.9 ([Y_2-H]$^-$), 810.8 ([Y_1-H]$^-$), 648.8 ([Y_0-H]$^-$)
Gb4Cer	1336.0 ([M-H]$^-$), 1134.8 ([Y_3-H]$^-$), 972.7 ([Y_2-H]$^-$), 810.7 ([Y_1-H]$^-$), 648.6 ([Y_0-H]$^-$)
GM3	1263.4 ([M-H]$^-$), 1245.8 ([M-H_2O-H]$^-$), 972.9 ([Y_2-H]$^-$), 810.8 ([Y_1-H]$^-$), 790.7 ([Z_1-H]$^-$), 648.8 ([Y_0-H]$^-$)
GM2	1466.9 ([M-H]$^-$), 1450.1 ([M-H_2O-H]$^-$), 1175.9 ([$Y_{2\beta}$-H]$^-$), 972.0 ([$Y_{2\alpha}$/$Y_{2\beta}$-H]$^-$), 811.0 ([Y_1-H]$^-$), 790.8 ([Z_1-H]$^-$), 648.8 ([Y_0-H]$^-$)
GM1	1629.0 ([M-H]$^-$), 1611.0 ([M-H_2O-H]$^-$), 1337.6 ([$Y_{2\beta}$-H^-]), 1319.5 ([$Z_{2\beta}$-H]$^-$), 1175.5 ([$Y_{3\alpha}$/$Y_{2\beta}$-H]$^-$), 1157.6 ($Y_{3\alpha}$/$Z_{2\beta}$ or $Z_{3\alpha}$/$Y_{2\beta}$-H]$^-$), 972.5 ([$Y_{2\alpha}$/$Y_{2\beta}$-H]$^-$), 810.3 ([Y_1-H]$^-$), 648.4 ([Y_0-H]$^-$)
GD3	720.9 ([M-2H]$^{2-}$), 1151.8 ([Y_3-H]$^-$), 860.6 ([Y_2-H]$^-$), 698.7 ([Y_1-H]$^-$), 581.1 ([B_2-H]$^-$), 289.8 ([B1-H])$^-$)
GD1a/ GD1b	959.2 ([M-2H]$^{2-}$), 1628.2 ([$Y_{4\alpha}$ or $Y_{2\beta}$-H]-), 1610.8 ([$Z_{4\alpha}$ or $Z_{2\beta}$-H]$^-$), 1338.4 ([$Y_{4\alpha}$/$Y_{2\beta}$-H]$^-$), 1173.8 ([$Y_{3\alpha}$/$Y_{2\beta}$-H]$^-$), 970.6 ([$Y_{2\alpha}$/$Y_{2\beta}$-H]$^-$), 289.8 ([$B_{1\alpha}$ or $B_{1\beta}$-H]$^-$)

Supplemental Table S1B. List of GSL structures in UtE cells by LC-MS or MS/MS

**Proposed GSLs**	**Fragments**
GlcCer	810.7 ([M-H]$^-$), 648.6 ([Y_0-H]$^-$)
LacCer	970.8 ([M-H]$^-$), 808.8 ([Y_1-H]$^-$), 646.8 ([Y_0-H]$^-$)
Gb3Cer	1022.7 ([M-H]$^-$), 1004.8 ([M-H_2O-H]$^-$), 860.8 ([Y_2-H]$^-$), 842.8 ([Z_2-H]$^-$), 698.7 ([Y_1-H]$^-$), 536.5 ([Y_0-H]$^-$)
Gb4Cer	1225.8 ([M-H]$^-$), 1207.8 ([M-H_2O-H]$^-$), 1022.7 ([Y_3-H]$^-$),1004.8 ([Z_3-H]$^-$), 860.4 ([Y_2-H]$^-$), 842.6 ([Z_2-H]$^-$), 698.4 ([Y_1-H]$^-$), 679.3 ([Z_1-H]$^-$), 535.5 ([Y_0-H]$^-$)
GM3	1263.3 ([M-H]$^-$), 1246.0 ([M-H_2O-H]$^-$), 972.8 ([Y_2-H]$^-$), 810.6 ([Y_1-H]$^-$), 792.8 ([Z_1-H]$^-$), 648.8 ([Y_0-H]$^-$)
GM2	1466.6 ([M-H]$^-$), 1450.1 ([M-H_2O-H]$^-$), 1175.9 ([$Y_{2\beta}$-H]$^-$), 970.9 ([$Y_{2\alpha}$/$Y_{2\beta}$-H]$^-$), 810.8 ([Y_1-H]$^-$), 792.8 ([Z_1-H]$^-$), 648.6 ([Y_0-H]$^-$)
GM1	1628.4 ([M-H]$^-$), 1611.0 ([M-H_2O-H]$^-$), 1338.0 ([$Y_{2\beta}$-H$^-$]), 1176.0 ([$Y_{3\alpha}$/$Y_{2\beta}$-H]$^-$), 972.8 ([$Y_{2\alpha}$/$Y_{2\beta}$-H]$^-$), 809.7 ([Y_1-H]$^-$), 648.9 ([Y_0-H]$^-$)
GD3	1444.1 ([M-H]$^-$), 1424.9 ([M-H_2O-H]$^-$), 1152.0 ([Y_3-H]$^-$), 1134.7 ([Z_3-H]$^-$), 860.7 ([Y_2-H]$^-$), 699.8 (Y_1-H]$^-$), 581.2 ([B_2-H]$^-$)
GD1a/ GD1b	959.2 ([M-2H]$^{2-}$), 1628.1 ([$Y_{4\alpha}$ or $Y_{2\beta}$-H]$^-$), 1610.0 ([$Z_{4\alpha}$ or $Z_{2\beta}$-H]$^-$), 1337.8 ([$Y_{4\alpha}$/$Y_{2\beta}$-H]$^-$), 973.6 ($Y_{2\alpha}$/$Y_{2\beta}$-H)$^-$), 811.0 ([Y_1-H]$^-$), 647.8 (Y_0-H)$^-$), 289.8 ([$B_{1\alpha}$ or $B_{1\beta}$-H]$^-$)

Supplemental Table S1C. List of GSL structures in MRC-iPSCs by LC-MS or MS/MS

Proposed GSLs	Fragments
GlcCer	698.3 ($[M-H]^-$), 536.3 ($[Y_0-H]^-$)
LacCer	860.3 ($[M-H]^-$), 698.3 ($[Y_1-H]^-$), 536.2 ($[Y_0-H]^-$)
Gb3Cer	1022.3 ($[M-H]^-$), 860.3 ($[Y_2-H]^-$), 698.3 ($[Y_1-H]^-$), 536.4 ($[Y_0-H]^-$)
Gb4Cer	1225.4 ($[M-H]^-$), 1022.3 ($[Y_3-H]^-$), 860.4 ($[Y_2-H]^-$), 698.3 ($[Y_1-H]^-$), 536.4 ($[Y_0-H]^-$)
Gb5Cer	1387.5 ($[M-H]^-$), 1225.4 ($[Y_4-H]^-$), 1207.3 ($[Z_4-H]^-$), 1022.4 ($[Y_3-H]^-$), 1004.3 ($[Z_3-H]^-$), 860.4 ($[Y_2-H]^-$), 698.3 ($[Y_1-H]^-$), 536.2 ($[Y_0-H]^-$)
Fucosyl-Gb5Cer	1533.5 ($[M-H]^-$), 1369.6 ($[Z_5-H]^-$), 1225.7 ($[Y_4-H]^-$), 1022.4 ($[Y_3-H]^-$), 860.3 ($[Y_2-H]^-$), 698.3 ($[Y_1-H]^-$), 536.3 ($[Y_0-H]^-$)
Sialyl-Gb5Cer	1678.8 ($[M-H]^-$), 1661.0 ($[M-H_2O-H]^-$), 1387.9 ($[Y_5-H]^-$), 1369.8 ($[Z_5-H]^-$), 1225.8 ($[Y_4-H]^-$), 1207.6 ($[Z_4-H]^-$), 1022.7 ($[Y_3-H]^-$), 1004.8 ($[Z_3-H]^-$), 860.7 ($[Y_2-H]^-$), 842.5 ($[Z_2-H]^-$), 698.6 ($[Y_1-H]^-$), 680.6 ($[Z_1-H]^-$), 536.6 ($[Y_0-H]^-$), 518.6 ($[Z_0-H]^-$)
(n)Lc4Cer	1225.7 ($[M-H]^-$), 1063.4 ($[Y_4-H]^-$), 1045.4 ($[Z_3-H]^-$), 860.3 ($[Y_2-H]^-$), 698.3 ($[Y_1-H]^-$), 536.3 ($[Y_0-H]^-$)
IV Fucosyl-(n)Lc4Cer	1371.4 ($[M-H]^-$), 1207.4 ($[Z_4-H]^-$), 1063.4 $[Y_3-H]^-$), 1045.4 ($[Z_3-H]^-$), 860.2 ($[Y_2-H]^-$), 698.2 ($[Y_1-H]^-$), 536.4 ($[Y_0-H]^-$)
GM3	1151.7 ($[M-H]^-$), 860.7 ($[Y_2-H]^-$), 698.5 ($[Y_1-H]^-$), 536.2 ($[Y_0-H]^-$), 518.5 ($[Z_0-H]^-$)
GM2	1354.8 ($[M-H]^-$), 1063.6 ($[Y_{2\beta}-H]^-$), 860.6 ($[Y_{2\alpha}/Y_{2\beta}-H]^-$), 698.6 ($[Y_1-H]^-$), 680.6 ($[Z_1-H]^-$), 536.5 ($[Y_0-H]^-$), 518.4 ($[Z_0-H]^-$)
GM1	1516.8 ($[M-H]^-$), 1499.0 ($[M-H_2O-H]^-$), 1225.8 ($[Y_4-H]^-$), 1063.8 ($[Y_{3\alpha}/Y_{2\beta}-H]^-$), 1045.8 ($[Y_{3\alpha}/Z_{2\beta}$ or $Z_{3\alpha}/Y_{2\beta}-H]^-$), 860.6 ($[Y_{2\alpha}/Y_{2\beta}-H]^-$), 698.6 ($[Y_1-H]^-$), 680.6 ($[Z_1-H]^-$), 536.5 ($[Y_0-H]^-$)
GD3	720.9($[M-2H]^{2-}$), 1151.8 ($[Y_3-H]^-$), 1133.5 $(Z_3-H)^-$), 860.6 ($[Y_2-H]^-$), 698.6 $(Y_1-H)^-$), 581.3 ($[B_2-H]^-$), 536.5 ($[Y_0-H]^-$), 290.0 ($[B_1-H]^-$)
GD2	822.4 $(M-2H)^{2-}$), 1354.9 ($[Y_{3\alpha}$ or $Y_{2\beta}-H]^-$), 1063.8 ($[Y_{3\alpha}/Y_{2\beta}-H]^-$), 861.4 ($[Y_{2\alpha}/Y_{2\beta}-H]^-$), 698.4 ($[Y_1-H]^-$), 582.1 ($[B_2-H]^-$), 536.4 ($[Y_0-H]^-$), 290.0 ($[B_1-H]^-$)

Supplemental Table S1D. List of GSL structures in UtE-iPSCs by LC-MS or MS/MS

**Proposed GSLs**	**Fragments**
GlcCer	698.3 ($[M\text{-}H]^-$), 536.4 ($[Y_0\text{-}H]^-$)
LacCer	860.3 ($[M\text{-}H]^-$), 698.3 ($[Y_1\text{-}H]^-$), 536.4 ($[Y_0\text{-}H]^-$)
Gb3Cer	1022.4 ($[M\text{-}H]^-$), 860.4 ($[Y_2\text{-}H]^-$), 698.3 ($[Y_1\text{-}H]^-$), 536.2 ($[Y_0\text{-}H]^-$)
Gb4Cer	1225.4 ($[M\text{-}H]^-$), 1022.4 ($[Y_3\text{-}H]^-$), 860.3 ($[Y_2\text{-}H]^-$), 698.3 ($[Y_1\text{-}H]^-$), 536.3 ($[Y_0\text{-}H]^-$)
Gb5Cer	1387.4 ($[M\text{-}H]^-$), 1225.5 ($[Y_4\text{-}H]^-$), 1207.6 ($[Z_4\text{-}H]^-$), 1022.3 ($[Y_3\text{-}H]^-$), 1004.3 ($[Z_3\text{-}H]^-$), 860.3 ($[Y_2\text{-}H]^-$), 698.3 ($[Y_1\text{-}H]^-$), 536.4 ($[Y_0\text{-}H]^-$)
Fucosyl-Gb5Cer	1533.4 ($[M\text{-}H]^-$), 1387.4 ($[Y_5\text{-}H]^-$), 1225.5 ($[Y_4\text{-}H]^-$), 1022.4 ($[Y_3\text{-}H]^-$), 698.4 ($[Y_1\text{-}H]^-$), 536.6 ($[Y_0\text{-}H]^-$), 1207.3 ($[Z_4\text{-}H]^-$)
Sialyl-Gb5Cer	1678.8 ($[M\text{-}H]^-$), 1661.0 ($[M\text{-}H_2O\text{-}H]^-$), 1387.9 ($[Y_5\text{-}H]^-$), 1370.0 ($[Z_5\text{-}H]^-$), 1225.9 ($[Y_4\text{-}H]^-$), 1207.9 ($[Z_4\text{-}H]^-$), 1022.7 ($[Y_3\text{-}H]^-$), 1004.7 ($[Z_3\text{-}H]^-$), 860.7 ($[Y_2\text{-}H]^-$), 842.7 ($[Z_2\text{-}H]^-$), 698.6 ($[Y_1\text{-}H]^-$), 680.6 ($[Z_1\text{-}H]^-$), 536.5 ($[Y_0\text{-}H]^-$)
(n)Lc4Cer	1225.7 ($[M\text{-}H]^-$), 1045.3 ($[Z_3\text{-}H]^-$), 860.3 ($[Y_2\text{-}H]^-$), 698.3 ($[Y_1\text{-}H]^-$), 536.3 ($[Y_0\text{-}H]^-$), 1063.3 ($[Y_3\text{-}H]^-$)
IV Fucosyl-(n)Lc4Cer	1371.4 ($[M\text{-}H]^-$), 1207.4 ($[Z_4\text{-}H]^-$), 1063.6 $[Y_3\text{-}H]^-$), 1045.2 ($[Z_3\text{-}H]^-$), 860.4 ($[Y_2\text{-}H]^-$), 698.4 ($[Y_1\text{-}H]^-$), 536.3 ($[Y_0\text{-}H]^-$)
GM3	1151.7 ($[M\text{-}H]^-$), 860.8 ($[Y_2\text{-}H]^-$), 698.6 ($[Y_1\text{-}H]^-$), 536.5 ($[Y_0\text{-}H]^-$)
GM2	1354.8 ($[M\text{-}H]^-$), 1063.6 ($[Y_{2\beta}\text{-}H]^-$), 860.6 ($[Y_{2\alpha}/Y_{2\beta}\text{-}H]^-$), 698.5 ($[Y_1\text{-}H]^-$), 536.3 ($[Y_0\text{-}H]^-$)
GM1	1516.8 ($[M\text{-}H]^-$), 1498.9 ($[M\text{-}H_2O\text{-}H]^-$), 1225.8 ($[Y_4\text{-}H]^-$), 1063.8 ($[Y_{3\alpha}/Y_{2\beta}\text{-}H]^-$), 1045.8 ($[Y_{3\alpha}/Z_{2\beta}$ or $Z_{3\alpha}/Y_{2\beta}\text{-}H]^-$), 860.6 ($[Y_{2\alpha}/Y_{2\beta}\text{-}H]^-$), 698.5 ($[Y_1\text{-}H]^-$), 680.6 ($[Z_1\text{-}H]^-$), 536.5 ($[Y_0\text{-}H]^-$)
GD3	720.9($[M\text{-}2H]^{2-}$), 1151.7 ($[Y_3\text{-}H]^-$), 860.5 ($[Y_2\text{-}H]^-$), 698.6 ($Y_1\text{-}H)^-$), 581.2 ($[B_2\text{-}H]^-$), 536.6 ($[Y_0\text{-}H]^-$), 290.0 ($[B_1\text{-}H]^-$)

Supplemental Table S2A. List of GSL structures in MRC-iPS-NSCs by LC-MS or MS/MS

**Proposed GSLs**	**Fragments**
GlcCer	698.5 ($[M-H]^-$), 536.4 ($[Y_0-H]^-$)
LacCer	860.6 ($[M-H]^-$), 698.4 ($[Y_1-H]^-$), 680.5 ($[Z_1-H]^-$), 536.5 ($[Y_0-H]^-$)
Gb3Cer	1022.8 ($[M-H]^-$), 860.6 ($[Y_2-H]^-$), 698.5 ($[Y_1-H]^-$), 536.2 ($[Y_0-H]^-$), 518.4 ($[Z_0-H]^-$)
Gb4Cer	1225.7 ($[M-H]^-$), 1183.8 ([M-Ac-H]-), 1022.5 ($[Y_3-H]^-$), 860.6 ($[Y_2-H]^-$), 842.6 ($[Z_2-H]^-$), 698.5 ($[Y_1-H]^-$), 680.5 ($[Z_1-H]^-$), 536.4 ($[Y_0-H]^-$), 518.3 ($[Z_0-H]^-$)
Lc3Cer	1063.8 ($[M-H]^-$), 860.7 ($[Y_2-H]^-$), 842.6 ($[Z_2-H]^-$), 698.6 ($[Y_1-H]^-$), 536.5 ($[Y_0-H]^-$)
(n)Lc4Cer	1225.7 ($[M-H]^-$), 1183.8 ($[M-Ac-H]^-$), 1063.8 ($[Y_3-H]^-$), 1045.8 ($[Z_3-H]^-$), 860.6 ($[Y_2-H]^-$), 842.6 ($[Z_2-H]^-$), 698.5 ($[Y_1-H]^-$), 680.5 ($[Z_1-H]^-$), 535.4 ($[Y_0-H]^-$), 518.3 ($[Z_0-H]^-$)
**III Fucosyl-(n)Lc4Cer**	1371.9 ($[M-H]^-$), 1209.8 ($[Y_{3\alpha}-H]^-$), 1045.7 ($[Y_{3\beta}/Z_{3\alpha}$ or $Z_{3\beta}/Y_{3\alpha}-H]^-$), 860.6 ($[Y_2-H]^-$), 842.5 ($[Z_2-H]^-$), 698.3 ($[Y_1-H]^-$), 536.3 ($[Y_0-H]^-$), 510.2 ($[B_2-H]^-$)
GM3	1151.7 ($[M-H]^-$), 1133.8 ($[M-H_2O-H]^-$), 860.6 ($[Y_2-H]^-$), 698.6 ($[Y_1-H]^-$), 680.4 ($[Z_1-H]^-$), 536.5 ($[Y_0-H]^-$)
GM2	1354.8 ($[M-H]^-$), 1336.9 ($[M-H_2O-H]^-$), 1063.7 ($[Y_{2\beta}-H]^-$), 860.6 ($[Y_{2\alpha}/Y_{2\beta}$-H]-), 698.7 ($[Y_1-H]^-$), 680.5 ($[Z_1-H]^-$), 536.3 ($[Y_0-H]^-$)
GM1	1516.8 ($[M-H]^-$), 1499.0 ($[M-H_2O-H]^-$), 1225.8 ($[Y_{2\beta}-H]^-$), 1207.8 ($[Z_{2\beta}-H]^-$), 1063.7 ($[Y_{3\alpha}/Y_{2\beta}-H]^-$), 1045.7 ($[Y_{3\alpha}/Z_{2\beta}$ or $Z_{3\alpha}/Y_{2\beta}-H]^-$), 860.6 ($[Y_{2\alpha}/Y_{2\beta}$-H]-), 842.7 ($[Z_{2\alpha}/Y_{2\beta}$ or $Y_{2\alpha}/Z_{2\beta}-H]^-$), 698.5 ($[Y_1-H]^-$), 536.5 ($[Y_0-H]^-$)
GD3	720.9 ($[M-2H]^{2-}$), 1151.7 ($[Y_3-H]^-$), 1133.8 ($[Z_3$-H]-), 860.7 ($[Y_2-H]^-$), 698.4 ($[Y_1-H]^-$), 680.6 ($[Z_1-H]^-$), 581.1 ($[B_2-H]^-$), 536.5 ($[Y_0-H]^-$), 519.4 ($[Z_0-H]^-$), 290.0 ($[B_1-H]^-$)
GD2	822.4 ($[M-2H]^{2-}$), 1354.9 ($[Y_{3\alpha}-H]^-$), 1337.9 ($[Z_{3\alpha}-H]^-$), 1063.9 ($[Y_{2\alpha}-H]^-$), 860.7 ($[Y_{2\alpha}/Y_{2\beta}-H]^-$), 698.5 ($[Y_1-H]^-$), 680.7 ($[Z_1-H]^-$), 581.2 ($[B_2-H]^-$), 536.3 ($[Y_0-H]^-$), 289.9 ($[B_{1\alpha}-H]^-$)
GD1a/ GD1b	903.5 ($[M-2H]^{2-}$), 1516.9 ($[Y_{4\alpha}$or $Y_{2\beta}-H]^-$), 1355.1 ($[Y_{3\alpha}-H]^-$), 1225.8 ($[Y_{4\alpha}/Y_{2\beta}-H]^-$), 860.8 ($[Y_{2\alpha}/Y_{2\beta}-H]^-$), 289.9 ($[B_{1\alpha}$ or $B_{1\beta}-H]^-$)

Supplemental Table S2B. List of GSL structures in UtE-iPS-NSCs by LC-MS or MS/MS

**Proposed GSLs**	**Fragments**
GlcCer	698.5 ($[M-H]^-$), 536.6 ($[Y_0-H]^-$)
LacCer	860.7 ($[M-H]^-$), 698.7 ($[Y_1-H]^-$), 680.5 ($[Z_1-H]^-$), 536.5 ($[Y_0-H]^-$)
Gb3Cer	1022.8 ($[M-H]^-$), 860.6 ($[Y_2-H]^-$), 698.7 ($[Y_1-H]^-$), 536.5 ($[Y_0-H]^-$)
Gb4Cer	1225.7 ($[M-H]^-$), 1183.8 ($M-Ac-H]^-$), 1023.3 ($[Y_3-H]^-$), 860.7 ($[Y_2-H]^-$), 842.6 ($[Z_2-H]^-$), 698.6 ($[Y_1-H]^-$), 536.4 ($[Y_0-H]^-$)
Lc3Cer	1063.7 ($[M-H]^-$), 698.7 ($[Y_1-H]^-$), 536.5 ($[Y_0-H]^-$)
(n)Lc4Cer	1225.7 ($[M-H]^-$), 1183.8 ($[M-Ac-H]^-$), 1063.7 ($[Y_3-H]^-$), 1045.9 ($[Z_3-H]^-$), 860.7 ($[Y_2-H]^-$), 842.6 ($[Z_2-H]^-$), 698.6 ($[Y_1-H]^-$), 536.4 ($[Y_0-H]^-$)
**III Fucosyl-(n)Lc4Cer**	1371.8 ($[M-H]^-$), 1208.9 ($[Y_{3\alpha}-H]^-$), 1045.8 ($[Y_{3\beta}/Z_{3\alpha}$ or $Z_{3\beta}/Y_{3\alpha}-H]^-$), 860.7 ($[Y_2-H]^-$), 842.7 ($[Z_2-H]^-$)698.8 ($[Y_1-H]^-$), 536.6 ($[Y_0-H]^-$)
GM3	1151.7 ($[M-H]^-$), 1132.6 ($[M-H_2O-H]^-$), 860.7 ($[Y_2-H]^-$), 843.2 ($[Z_2-H]^-$), 698.5 ($[Y_1-H]^-$), 680.7 ($[Z_1-H]^-$), 536.5 ($[Y_0-H]^-$)
GM2	1354.8 ($[M-H]^-$), 1336.8 ($[M-H_2O-H]^-$), 1063.8 ($[Y_{2\alpha}-H]^-$), 860.6 ($[Y_{2\alpha}/Y_{2\beta}-H]^-$), 698.6 ($[Y_1-H]^-$), 536.5 ($[Y_0-H]^-$)
GM1	1516.8 ($[M-H]^-$), 1499.0 ($[M-H_2O-H]^-$), 1225.8 ($[Y_4-H]^-$), 1063.9 ($[Y_{3\alpha}/Y_{2\beta}-H]^-$), 1045.8 ($[Y_{3\alpha}/Z_{2\beta}$ or $Z_{3\alpha}/Y_{2\beta}-H]^-$), 860.7 ($[Y_{2\alpha}/Y_{2\beta}-H]^-$), 842.7 ($[Z_{2\alpha}/Y_{2\beta}$ or $Z_{2\beta}/Y_{2\alpha}-H]^-$), 698.6 ($[Y_1-H]^-$), 536.6 ($[Y_0-H]^-$)
GD3	720.9 ($[M-2H]^{2-}$), 1151.8 ($[Y_3-H]^-$), 1133.7 ($[Z_3-H]-$), 860.7 ($[Y_2-H]^-$), 698.5 ($[Y_1-H]^-$), 680.5 ($[Z_1-H]^-$), 581.2 ($[B_2-H]^-$), 563.2 ($[C_2-H_2O-H]^-$), 536.4 ($[Y_0-H]^-$), 290.0 ($[B_1-H]^-$)
GD2	822.4 ($[M-2H]^{2-}$), 1354.9 ($[Y_{3\alpha}-H]^-$), 1063.7 ($[Y_{2\alpha}-H]^-$), 860.6 ($Y_{2\alpha}/Y_{2\beta}-H]^-$), 581.1 ($[B_2-H]^-$), 563.1 ($[C_3-H_2O-H]^-$), 536.1 ($[Y_0-H]^-$), 290.0 ($[B_{1\alpha}-H]^-$)
GD1a/ GD1b	903.5 ($[M-2H]^{2-}$), 1517.0 ($[Y_{4\alpha}$or $Y_{2\beta}-H]^-$), 1226.0 ($[Y_{4\alpha}/Y_{2\beta}-H]^-$), 860.7 ($[Y_{2\alpha}/Y_{2\beta}-H]^-$), 843.1 ($[Y_{2\alpha}/Z_{2\beta}$ or $Z_{2\alpha}/Y_{2\beta}-H]^-$), 698.3 ($[Y_1-H]^-$), 581.3 ($[B_{1\alpha}$ or $B_{1\beta}-H]^-$), 289.8 ($[B_{1\alpha}$ or $B_{1\beta}-H]^-$)

Supplemental Table S2C. List of GSL structures in MRC-iPS-EB outgrowth by LC-MS or MS/MS

Proposed GSLs	Fragments
GlcCer	698.6 ($[M-H]^-$), 536.5 ($[Y_0-H]^-$)
LacCer	860.6 ($[M-H]^-$), 698.6 ($[Y_1-H]^-$), 680.4 ($[Z_1-H]^-$), 536.5 ($[Y_0-H]^-$)
Gb3Cer	1022.7 ($[M-H]^-$), 1004.7 ($[M-H_2O-H]^-$), 860.6 ($[Y_2-H]^-$), 698.5 ($[Y_1-H]^-$), 680.4 ($[Z_1-H]^-$), 536.6 ($[Y_0-H]^-$)
Gb4Cer	1225.8 ($[M-H]^-$), 1022.7 ($[Y_3-H]^-$), 860.5 ($[Y_2-H]^-$), 698.3 ($[Y_1-H]^-$), 681.1 ($[Z_1-H]^-$)
Lc3Cer	1063.7 ($[M-H]^-$), 860.6 ($[Y_2-H]^-$), 698.5 ($[Y_1-H]^-$), 536.5($[Y_0-H]^-$)
(n)Lc4Cer	1225.8 ($[M-H]^-$), 1063.3 ($[Y_3-H]^-$), 860.5 ($[Y_2-H]^-$), 698.3 ($[Y_1-H]^-$), 681.1 ($[Z_1-H]^-$)
**III Fucosyl-(n)Lc4Cer**	1371.9 ($[M-H]^-$), 1225.8 ($[Y_{3\beta}-H]^-$), 1209.7 ($[Y_{3\alpha}-H]^-$), 1045.8 ($[Y_{3\beta}/Z_{3\alpha}$ or $Z_{3\beta}/Y_{3\alpha}-H]^-$), 860.6 ($[Y_2-H]^-$), 842.5 ($[Z_2-H]^-$), 698.6 ($[Y_1-H]^-$), 680.4 ($[Z_1-H]^-$), 536.5 ($[Y_0-H]^-$), 510.1 ($[B_2-H]^-$)
GM3	1151.7 ($[M-H]^-$), 860.6 ($[Y_2-H]^-$), 698.6 ($[Y_1-H]^-$), 680.4 ($[Z_1-H]^-$), 536.5 ($[Y_0-H]^-$), 518.6 ($[Z_0-H]^-$),
GM2	1354.8 ($[M-H]^-$), 1063.8 ($[Y_{2\beta}-H]^-$), 860.7 ($[Y_{2\alpha}/Y_{2\beta}$-H]-), 698.6 ($[Y_1-H]^-$), 680.5 ($[Z_1-H]^-$), 536.6 ($[Y_0-H]^-$)
GM1	1516.8 ($[M-H]^-$), 1499.0 ($[M-H_2O-H]^-$), 1225.9 ($[Y_{2\beta}-H]^-$), 1063.8 ($[Y_{3\alpha}/Y_{2\beta}-H]^-$), 1045.8 ($[Y_{3\alpha}/Z_{2\beta}$ or $Z_{3\alpha}/Y_{2\beta}-H]^-$), 860.7 ($[Y_{2\alpha}/Y_{2\beta}-H]^-$), 842.6 ($[Z_{2\alpha}/Y_{2\beta}$ or $Y_{2\alpha}/Z_{2\beta}-H]^-$), 698.6 ($[Y_1-H]^-$), 680.5($[Z_1-H]^-$), 536.6 ($[Y_0-H]^-$)
GD3	720.9 ($M-2H]^{2-}$), 1151.8 ($[Y_3-H]^-$), 1133.9 ($[Z_3$-H]-), 860.7 ($[Y_2-H]^-$), 698.6 ($[Y_1-H]^-$), 680.5 ($[Z_1-H]^-$), 581.2 ($[B_2-H]^-$), 536.5 ($[Y_0-H]^-$), 290.0 ($[B_1-H]^-$)
GD2	822.4 ($[M-2H]^{2-}$), 1354.9 ($[Y_{3\alpha}-H]^-$), 1336.9 ($Z_{3\alpha}-H]^-$), 1063.8 ($[Y_{2\alpha}-H]^-$), 1045.8 ($[Z_{2\alpha}-H]^-$), 860.7 ($Y_{2\alpha}/Y_{2\beta}-H]^-$), 698.5 ($[Y_1-H]^-$), 680.8 ($[Z_1-H]^-$), 581.2 ($[B_{2\alpha}-H]^-$), 536.5 ($[Y_0-H]^-$), 290.0 ($[B_{1\alpha}-H]^-$)
GD1a/ GD1b	903.50 ($[M-2H]^{2-}$), 1516.9 ($[Y_{4\alpha}$ or $Y_{2\beta}-H]^-$), 1499.0 ($Z_{4\alpha}$ or $Z_{2\beta}-H]^-$), 1355.0 ($[Y_{3\alpha}-H]^-$), 1225.9 ($[Y_{4\alpha}/Y_{2\beta}-H]^-$), 1151.9 ($[Y_{2\alpha}-H]^-$),1063.6 ($[Y_{3\alpha}/Y_{2\beta}-H]^-$), 860.6 ($[Y_{2\alpha}/Y_{2\beta}-H]^-$), 843.3 ($[Y_{2\alpha}/Z_{2\beta}$ or $Z_{2\alpha}/Y_{2\beta}-H]^-$), 698.5 ($Y_1-H]^-$), 680.6 ($[Z_1-H]^-$), 536.5 ($[Y_0-H]^-$), 289.9 ($[B_{1\alpha}$ or $B_{1\beta}-H]^-$)

Supplemental Table S2D. List of GSL structures in UtE-iPS-EB outgrowth by LC-MS or MS/MS

**Proposed GSLs**	**Fragments**
GlcCer	698.5 ([M-H]$^-$), 536.4 ([Y_0-H]$^-$)
LacCer	860.6 ([M-H]$^-$), 698.5 ([Y_1-H]$^-$), 680.5 ([Z_1-H]$^-$), 536.5 ([Y_0-H]$^-$)
Gb3Cer	1022.7 ([M-H]$^-$), 1004.8 ([M-H_2O-H]$^-$), 860.6 ([Y_2-H]$^-$), 698.5 ([Y_1-H]$^-$), 680.6 ([Z_1-H]$^-$), 536.5 ([Y_0-H]$^-$)
Gb4Cer	1225.8 ([M-H]$^-$), 1206.8 (M-H_2O-H]$^-$), 1022.6 ([Y_3-H]$^-$), 860.7 ([Y_2-H]$^-$), 842.7 ([Z_2-H]$^-$), 698.5 ([Y_1-H]$^-$), 680.7 ([Z_1-H]$^-$), 536.5 ([Y_0-H]$^-$), 518.4 ([Z_0-H]$^-$)
Lc3Cer	1063.7 ([M-H]$^-$), 860.5 ([Y_2-H]$^-$), 698.6 ([Y_1-H]$^-$)
(n)Lc4Cer	1225.7 ([M-H]$^-$), 1206.8 ([M-H_2O-H]$^-$), 1063.7 ([Y_3-H]$^-$), 1045.8 ([Z_3-H]$^-$), 860.7 ([Y_2-H]$^-$), 842.7 ([Z_2-H]$^-$), 698.5 ([Y_1-H]$^-$), 680.7 ([Z_1-H]$^-$), 536.5 ([Y_0-H]$^-$), 518.4 ([Z_0-H]$^-$)
**III Fucosyl-(n)Lc4Cer**	1371.8 ([M-H]$^-$), 1209.9 ([$Y_{3\alpha}$-H]$^-$), 1207.6 ([$Z_{3\beta}$-H]$^-$), 1045.9 ([$Y_{3\beta}$/$Z_{3\alpha}$ or $Z_{3\beta}$/$Y_{3\alpha}$-H]$^-$), 860.7 ([Y_2-H]$^-$), 698.6 ([Y_1-H]$^-$), 680.6 ([Z_1-H]$^-$), 536.4 ([Y_0-H]$^-$)
GM3	1151.7 ([M-H]$^-$), 860.8 ([Y_2-H]$^-$), 698.5 ([Y_1-H]$^-$), 680.6 ([Z_1-H]$^-$), 536.5 ([Y_0-H]$^-$), 518.5 ([Z_0-H]$^-$),
GM2	1354.8 ([M-H]$^-$), 1063.7 ([$Y_{2\beta}$-H]$^-$), 860.6 ([$Y_{2\alpha}$/$Y_{2\beta}$-H]-), 698.5 ([Y_1-H]$^-$), 680.5 ([Z_1-H]$^-$), 536.5 ([Y_0-H]$^-$)
GM1	1516.8 ([M-H]$^-$), 1498.9 ([M-H_2O-H]$^-$), 1225.8 ([Y_2-H]$^-$), 1063.8 ([$Y_{3\alpha}$/$Y_{2\beta}$-H]$^-$), 1045.8 ([$Y_{3\alpha}$/$Z_{2\beta}$ or $Z_{3\alpha}$/$Y_{2\beta}$-H]$^-$), 860.6 ([$Y_{2\alpha}$/$Y_{2\beta}$-H]-), 842.6 ([$Z_{2\alpha}$/$Y_{2\beta}$ or $Y_{2\alpha}$/$Z_{2\beta}$-H]$^-$), 698.6 ([Y_1-H]$^-$), 680.5 ([Z_1-H]$^-$), 536.5 ([Y_0-H]$^-$)
GD3	720.9 (M-2H]$^{2-}$), 1151.8 ([Y_3-H]$^-$), 1134.7 ([Z_3-H]-), 860.7 ([Y_2-H]$^-$), 698.6 ([Y_1-H]$^-$), 680.6 ([Z_1-H]$^-$), 581.1 ([B_2-H]$^-$), 536.4 ([Y_0-H]$^-$), 290.0 ([B_1-H]$^-$)
GD2	822.4 ([M-2H]$^{2-}$), 1354.9 ([$Y_{3\alpha}$-H]$^-$), 1063.8 ([$Y_{2\alpha}$-H]$^-$), 860.4 ($Y_{2\alpha}$/$Y_{2\beta}$-H]$^-$), 698.7 ([Y_1-H]$^-$), 680.7 ([Z_1-H]$^-$), 581.1 ([$B_{2\alpha}$-H]$^-$), 536.4 ([Y_0-H]$^-$), 290.1 ([$B_{1\alpha}$-H]$^-$)
GD1a/ GD1b	903.5 ([M-2H]$^{2-}$), 1516.9 ([$Y_{4\alpha}$or $Y_{2\beta}$-H]$^-$), 1498.9 ($Z_{4\alpha}$or $Z_{2\beta}$-H]$^-$), 1225.6 ([$Y_{4\alpha}$/$Y_{2\beta}$-H]$^-$), 1151.9 ([$Y_{2\alpha}$-H]$^-$), 1064.3 ([$Y_{3\alpha}$/$Y_{2\beta}$-H]$^-$), 860.5 ([$Y_{2\alpha}$/$Y_{2\beta}$-H]$^-$), 698.3 (Y_1-H]$^-$), 680.5 ([Z_1-H]$^-$), 536.6 ([Y_0-H]$^-$), 290.0 ([$B_{1\alpha}$ or $B_{1\beta}$-H]$^-$)
